# Diene-Extended Chalcones Enable Red Fluorescence Detection of Human Serum Albumin Through FA1 Binding

**DOI:** 10.3390/bios16100533

**Published:** 2026-09-24

**Authors:** Brian Corbin, Jessica Mason, Paityn Houglan, Amanda Murphey, Yi Pang

**Affiliations:** Department of Chemistry, The University of Akron, Akron, OH 44325, USA

**Keywords:** chalcone, human serum albumin (HSA), albumin sensing, fluorescent probes

## Abstract

Fluorescent molecular probes for selective and real-time detection of human serum albumin (HSA) protein in urine and blood have important applications in clinical diagnostics for various diseases. The majority of known fluorescent HSA probes give emission in the blue-green spectral region where biological autofluorescence could interfere with the detection. In this study, a series of chalcones with extended conjugation are shown to give red-emission with a large Stokes shift (λ_abs_ ≈ 430–445 nm, λ_em_ ≈ 620–640 nm), and low fluorescence in H_2_O. Upon addition of HSA protein, the fluorescence of these chalcones is shown to increase significantly, by as high as 30-fold. Potential site-selective binding to HSA has been examined by competitive displacement experiments using various known site-selective drugs. In conjunction with molecular docking experiments, the study further indicates that the chalcones could selectively enter the FA1 (Fatty Acid site 1) binding pocket (Drug Site III).

## 1. Introduction

Human serum albumin (HSA) is the most abundant plasma protein in human blood and plays a central role in maintaining osmotic pressure while serving as the primary transport protein for endogenous metabolites, fatty acids, hormones, and numerous pharmaceutical agents [1,2,3,4,5]. Owing to its central physiological role, abnormal HSA concentrations are closely associated with liver dysfunction, kidney disease, malnutrition, cardiovascular disorders, and systemic inflammation, making accurate HSA quantification an important tool in both clinical diagnostics and point-of-care testing [6,7,8,9]. Consequently, considerable effort has been devoted to the development of rapid, selective, and highly sensitive analytical platforms for HSA detection, with fluorescence-based biosensors emerging as particularly attractive candidates because of their simplicity, rapid response, and compatibility with point-of-care analysis [10,11,12,13,14,15,16].

Among the diverse analytical methods developed for HSA determination, fluorescence-based sensing has attracted particular interest because of its high sensitivity, excellent temporal resolution, operational simplicity, and compatibility with miniaturized and portable instrumentation [12,17]. Fluorescent sensors further enable non-destructive measurements using small sample volumes and can often provide real-time responses without extensive sample preparation [18]. Considerable effort has therefore been devoted toward developing small-molecule fluorophores capable of selectively recognizing HSA through fluorescence turn-on or wavelength-shifting mechanisms [19,20,21]. Although numerous fluorescent HSA probes have been reported, many rely on emission in the blue–green spectral region where biological autofluorescence can reduce sensitivity, while others exhibit limited site selectivity or provide little mechanistic insight into the molecular basis of protein recognition [12,14,17,19,22,23].

Donor–acceptor chalcones constitute an attractive class of environmentally responsive fluorophores owing to their straightforward synthesis, tunable intramolecular charge-transfer (ICT) character, and readily modifiable aromatic framework. Recently, our group reported a series of π-extended diene-conjugated chalcones exhibiting orange–red emission together with significantly improved fluorescence efficiencies relative to conventional chalcones through suppression of excited-state proton transfer pathways and enhancement of ICT [24]. The extended conjugation also shifts absorption and emission toward longer wavelengths where interference from endogenous fluorophores is reduced, making these molecules promising candidates for biological sensing applications [19,20,25,26,27]. Furthermore, systematic methoxy substitution provides a convenient platform for examining how subtle structural modifications influence protein recognition and fluorescence activation.

In this work, we investigate six π-extended diene chalcones as fluorescent probes for HSA recognition. The probes display pronounced fluorescence turn-on responses following HSA binding, accompanied by changes in both absorption and emission spectra consistent with formation of emissive protein-bound states. Through fluorescence titration, time-dependent measurements, competitive displacement experiments, and molecular docking studies, we establish clear substituent-dependent trends governing HSA recognition and demonstrate that these probes preferentially occupy the noncanonical FA1/hemin binding pocket rather than the classical Sudlow drug-binding sites. Unexpectedly, ligands occupying distant drug-binding sites modulate probe emission through long-range allosteric communication, demonstrating that fluorescence output is governed not solely by binding affinity but also by the protein conformational ensemble stabilized following ligand recognition. These findings establish π-extended chalcones as a versatile platform for fluorescent HSA sensing while providing new mechanistic insight into how ligand structure and protein dynamics collectively regulate fluorescence activation.

## 2. Materials and Methods

A general structure for chalcones **1a**–**1f** is shown in Scheme 1. The complete synthesis and characterization of chalcones **1a**–**1f** have been reported in our previous work [24].

All reagents and solvents used for synthesis, spectroscopic studies, as well as biological analytes including human serum albumin (HSA), were purchased from Sigma-Aldrich (Burlington, MA, USA) or Fisher Scientific (Hampton, NH, USA) and used without further purification.

UV–vis absorption spectra were recorded on an HP 8453 diode array spectrophotometer (Hewlett-Packard, Palo Alto, CA, USA) at 25 °C. Fluorescence measurements were carried out using a FluoroMax-4 spectrofluorometer (HORIBA Scientific, Edison, NJ, USA). Absolute fluorescence quantum yields were determined using a calibrated Horiba integrating sphere (No. 5570830005) manufactured by HORIBA Scientific. Sample absorbance at the excitation wavelength was maintained below 0.1.

Competitive binding studies with ibuprofen and warfarin were performed using 10 μM chalcone solutions and 50 μL of 5% HSA (18.3 µM) in PBS. Due to the strong visible absorption of hemin and methyl orange, these experiments were performed using 2.5 μM chalcone solutions and 12.5 μL of 5% HSA (4.69 µM).

Limits of detection (LOD) and quantification (LOQ) were calculated from fluorescence titration experiments performed using 2.5 μM chalcone solutions in PBS with incremental additions of HSA from a 100 μM stock solution. LOD and LOQ were determined from the linear region of the calibration curves using the 3σ/slope and 10σ/slope criteria, respectively, where σ represents the standard deviation of the blank measurements. Calibration curves and additional information for each sensor are provided in the Supporting Information.

Molecular docking studies were performed using AutoDock 4.2.6 to model ligand–protein interactions [28]. Crystal structures of native human serum albumin (PDB ID: 1AO6), were obtained from the RCSB Protein Data Bank. For each ligand, 10 independent docking runs were performed, and binding energies were extracted directly from AutoDock outputs. Ligand conformations were optimized using a global search algorithm. Final ligand conformations were visualized using PyMol 3.20a and ChimeraX 1.11.1 [29,30].

## 3. Results and Discussion

### 3.1. Sensor Design

The six chalcones investigated in this work were recently developed as a series of π-extended donor–acceptor fluorophores in which systematic methoxy substitution was used to tune intramolecular charge transfer while preserving a common molecular framework [24]. Previous spectroscopic, crystallographic, and computational studies demonstrated that subtle changes in the substitution pattern modulate backbone planarity, excited-state relaxation, and fluorescence efficiency, producing environmentally responsive fluorophores that emit in the orange–red spectral region. These well-defined structure–property relationships make the series particularly attractive for investigating how small structural perturbations influence protein recognition and fluorescence activation.

### 3.2. HSA Sensitivity

To evaluate the HSA sensing performance of the chalcone series, the photophysical properties of **1a**–**1f** were examined in water and phosphate-buffered saline (PBS) (Figure S1). Most derivatives exhibited pronounced changes in their absorption profiles in PBS relative to water, characterized by broadening of the absorption band and the emergence of a red-shifted tail, consistent with the formation of aggregate-like or preassociated sensor populations under buffered ionic conditions. This assignment was further supported by concentration-dependent absorption measurements in PBS (0.625–12.5 μM; Figure S2), which revealed progressive bathochromic shifts and band broadening for most derivatives, consistent with concentration-driven J-like aggregation. Sensor **1a** is shown as a representative example in Figure 1. In water, **1a** displays a well-defined absorption band centered near 430 nm, whereas in PBS, a broadened, red-shifted absorption peak is observed near 550 nm. These aggregate-like species are accompanied by weak fluorescence in both water and PBS, indicating that nonradiative relaxation pathways dominate in aqueous media.

Addition of HSA resulted in substantial changes in the absorption and emission profiles of the chalcone probes. Upon introduction of HSA, the absorption bands became narrower and more defined, consistent with a change in the local environment and redistribution of the fluorophores from heterogeneous aqueous populations into protein-associated states. More importantly, all chalcones exhibited pronounced fluorescence turn-on responses upon addition of HSA (Figure 1 and Figure S1), accompanied by hypsochromic emission shifts indicative of formation of emissive protein-bound species. Among the series, sensor **1a** displayed the largest enhancement, exhibiting a 31-fold increase in fluorescence intensity relative to PBS alone (Figure 1c). Notably, despite the reduced fluorescence observed in PBS, the emission spectra recorded in the presence of HSA closely resembled those obtained in water. This behavior suggests that HSA binding disrupts the nonemissive, aggregated species present under buffered conditions and stabilizes a more emissive protein-bound conformation.

Due to the large fluorescent enhancement in response to HSA, the quantum yields were determined for each sensor (Table S1). Most of the derivatives exhibited modest quantum yields between 0.080 and 0.184, except **1d**, which exhibited the highest quantum yield of the series of 0.334. The quantum yield of **1d** in the presence of HSA is notably higher than the highest quantum yield of **1d** in pure organic solvents (e.g., 0.245 in DMSO) that we reported previously, suggesting that HSA binding may provide enhanced stabilization of these molecules that is not possible by regular solvation [24].

After establishing the fluorescence turn-on response of the chalcone series toward HSA, the concentration-dependent sensing behavior was examined by fluorescence titration in PBS (Figure 2). The resulting titration profiles were analyzed using the Hill equation, and the extracted apparent binding parameters are summarized in Table 1. All six chalcones exhibited rapid fluorescence activation at low HSA concentrations, followed by saturation behavior consistent with formation of a protein-bound emissive state. However, distinct substituent-dependent differences were observed in both the apparent binding affinity (K) and the shape of the titration response.

Introduction of methoxy substituents on the A-ring significantly influenced the apparent binding behavior. Relative to unsubstituted **1a** (K = 2.54 µM), both the *ortho*-substituted derivative **1b** (K = 2.04 µM) and *para*-substituted derivative **1c** (K = 1.55 µM) exhibited enhanced apparent affinity toward HSA. The 2,4-diOMe derivative **1d** displayed a similarly strong response (K = 1.77 µM), whereas the more sterically substituted derivatives **1e** and **1f** exhibited substantially weaker apparent affinities of 5.44 and 4.45 µM, respectively. These results suggest that methoxy substitution can enhance HSA recognition, but that excessive *ortho* substitution ultimately becomes unfavorable.

The Hill coefficients also exhibited a distinct substituent dependence. While **1a** produced an approximately hyperbolic response (*n* = 1.00), derivatives **1b**–**1d** displayed larger Hill coefficients ranging from 1.48 to 1.55, indicative of a steeper fluorescence activation profile. Because the fluorescence response reflects both binding and photophysical changes within the protein environment, these values are interpreted as phenomenological descriptors of the sensing response rather than direct evidence of cooperative binding. Nevertheless, the similar Hill coefficients observed for **1b**–**1d** suggest that incorporation of methoxy groups promotes a common recognition or activation mechanism within the HSA binding pocket. It appears that these derivatives contain sufficient methoxy substitution to enhance interactions with HSA while avoiding the extensive steric congestion associated with multiple ortho substituents. In our previous studies of these chalcones, increasing ortho methoxy substitution was shown to promote steric interactions with both the carbonyl group and conjugated backbone, resulting in larger A-ring torsional angles and increased nonradiative deactivation pathways [24]. The intermediate substitution patterns present in **1b**–**1d** therefore appear to provide a favorable balance between protein recognition and preservation of an emissive ICT-active geometry.

The analytical sensitivity of the probes was also analyzed and despite the structural diversity of the chalcones, all six derivatives exhibited excellent linearity over the initial HSA concentration range (R^2^ = 0.987–0.998). The corresponding calibration curves used for determination of the LOD and LOQ values are provided in Figures S3–S8. All probes achieved nanomolar limits of detection, with **1b** achieving the lowest LOD of 11 nM. This sensitivity compares favorably with related fluorescent HSA sensors [12,31]. For example, a recently reported chalcone bearing a naphthyl A-ring exhibited an LOD of 23 nM [32]. Sensors **1c** and **1d** exhibited moderately higher LODs of 101 and 160 nM, respectively. Notably, every derivative within the series retained nanomolar analytical sensitivity despite systematic modification of the A-ring, demonstrating that structural tuning of the sensing mechanism does not compromise the excellent analytical performance of the π-extended chalcone scaffold.

Despite the excellent analytical performance observed across the series, the apparent binding affinities do not correlate directly with the magnitude of fluorescence enhancement observed under bolus addition conditions (Figure 1). For example, **1c** exhibited the strongest apparent affinity within the series, whereas **1a** and **1f** produced the largest fluorescence turn-on responses. This observation suggests that fluorescence enhancement is not governed solely by binding strength, but also by the nature of the emissive protein-bound state populated following recognition. Consequently, binding affinity and fluorescence output appear to be partially decoupled within this sensor series.

The individual titration spectra (Figures S9–S20) reveal that fluorescence enhancement occurs progressively throughout the binding process rather than through an abrupt two-state transition. The gradual evolution of both the absorption and emission profiles suggests that intermediate populations are sampled as HSA concentration increases, consistent with progressive disruption of aggregate-like species and formation of emissive protein-bound conformations. Importantly, the spectroscopic evolution observed during titration mirrors the PBS-to-HSA spectral changes shown in Figure 1, further supporting the hypothesis that HSA extracts the chalcones from weakly emissive aggregate-like states into highly emissive protein-bound populations.

Although the titration experiments provide information regarding the concentration-dependent fluorescence response of each sensor, they do not address whether the initially formed protein-bound complexes remain stable following recognition. To test the stability of the HSA-Sensor complex, a temporal evolution of the HSA-bound state was performed following HSA addition (Figure 3 and Figures S21–S26). The change in fluorescence was found to be dependent on substitution pattern. Derivatives lacking ortho methoxy substitution (**1a** and **1c**) exhibited gradual decreases in fluorescence intensity over time, whereas chalcones containing one or more ortho methoxy groups generally displayed small increases in emission before reaching a stable plateau. A plausible explanation is that ortho methoxy substitution influences the orientation of the chalcone scaffold within the HSA binding pocket, favoring bound conformations that better preserve the emissive ICT-active geometry. In contrast, the greater conformational freedom available to **1a** and **1c** may permit relaxation into alternative bound states with lower fluorescence efficiencies. Notably, derivatives **1b** and **1d** exhibited the smallest changes in fluorescence intensity following the initial response, indicating rapid stabilization of the HSA-bound state.

### 3.3. Drug Displacement and Competitive Binding Studies

To identify the HSA binding site occupied by the chalcone sensors, competitive binding studies were performed using four well-established site markers: warfarin (Sudlow Site I), ibuprofen (Sudlow Site II), methyl orange, and hemin. Whereas warfarin and ibuprofen selectively probe the two classical drug-binding sites of HSA, methyl orange has been reported to interact with multiple fatty acid binding regions and therefore serves as a general probe of fatty acid site occupancy. In contrast, hemin binds with high specificity to the FA1 (heme) binding pocket (Drug Site III), providing a more definitive probe for this region. Competitive binding profiles for all six chalcones are shown in Figure 4.

Distinct substituent-dependent responses were observed upon addition of the competing ligands. Hemin produced the largest decrease in fluorescence intensity for every chalcone investigated, while methyl orange also caused substantial fluorescence quenching across the series. Because hemin binds specifically to the FA1 (heme) binding pocket, these results are consistent with competitive displacement of the chalcones from HSA rather than simple fluorescence quenching. Once displaced from the protein, the chalcones are returned to the aqueous PBS environment where, as demonstrated in Figure 1, they exist predominantly in a weakly emissive aggregate-like state. The pronounced fluorescence loss following hemin addition therefore provides strong experimental support for localization of the chalcones within the FA1/heme binding region. The similar, although less site-specific, response observed with methyl orange provides additional evidence that the chalcones occupy drug site III of HSA.

In contrast to hemin and methyl orange, neither ibuprofen nor warfarin produced a uniform fluorescence response across the chalcone series. Instead, both the magnitude and direction of the fluorescence change depended strongly on the substitution pattern of the A-ring, indicating that methoxy substitution modulates the sensitivity of the protein-bound fluorophore to allosteric perturbation.

The unsubstituted derivative **1a** together with the mono-methoxy derivatives **1b** and **1c** exhibited fluorescence enhancement following addition of ibuprofen, with the para-methoxy derivative **1c** displaying the largest response, approaching a 100% increase in fluorescence intensity (Figure 4). Because ibuprofen binds at Sudlow site II rather than the proposed chalcone binding pocket, this enhancement is unlikely to arise from direct displacement. Instead, these results suggest that ibuprofen binding induces a positive allosteric effect that stabilizes a more emissive protein-bound conformational ensemble rather than directly displacing the chalcone, consistent with established models of allosteric communication in ligand-binding proteins [33]. The pronounced response of **1c** further suggests that the para-methoxy substituent provides sufficient rotational freedom about the A-ring to enable the ligand to adapt to the subtle structural rearrangements accompanying ibuprofen binding. This enhanced conformational flexibility would facilitate adoption of a more planar, highly emissive bound geometry, thereby maximizing intramolecular charge transfer. It is also plausible that the para-methoxy substituent promotes favorable interactions within the binding pocket that effectively anchor the ligand while still permitting rotational adjustment of the conjugated backbone, resulting in a larger fluorescence enhancement than observed for the other derivatives.

In contrast to ibuprofen, warfarin produced a markedly different response. While the unsubstituted derivative **1a** underwent substantial fluorescence quenching, consistent with partial displacement or destabilization of the bound state, the methoxy-substituted derivatives were largely unaffected, with **1d** exhibiting a modest fluorescence enhancement of approximately 40%. This fluorescent enhancement of **1d** towards warfarin is interesting since it has the highest quantum yield (0.334) upon HSA binding in the series and is the only derivative to produce a modest fluorescent enhancement towards warfarin. However, **1d** displayed the opposite behavior upon addition of ibuprofen, undergoing a slight decrease in fluorescence rather than the enhancement observed for the mono-methoxy derivatives. Because neither warfarin nor ibuprofen is expected to directly compete for the proposed chalcone binding pocket, these contrasting responses are unlikely to arise from competitive displacement. Instead, they suggest that ligand binding at Sudlow sites I and II propagates distinct allosteric conformational changes through the HSA structure, thereby altering the local environment surrounding the bound chalcone and shifting the equilibrium between protein-bound conformational microstates possessing different fluorescence efficiencies [33,34]. The diminished sensitivity of the methoxy-substituted derivatives to warfarin, together with the modest fluorescence enhancement observed for **1d**, further suggests that methoxy substitution stabilizes the protein-bound conformation while retaining sufficient conformational flexibility to respond to allosterically induced structural rearrangements.

The responses of the more sterically congested derivatives **1e** and **1f** further support a conformational origin for the observed allosteric modulation. Both derivatives exhibited only minor fluorescence changes upon addition of ibuprofen or warfarin, suggesting that dual ortho–methoxy substitution restricts the ability of the bound chalcone to adapt to ligand-induced conformational changes propagated from the classical Sudlow sites. Despite this reduced allosteric response, both derivatives remained highly susceptible to displacement by hemin and exhibited substantial fluorescence loss upon addition of methyl orange, indicating that increased steric congestion does not prevent binding within the FA1/hemin pocket but instead limits the conformational adaptability of the protein-bound sensor. Collectively, these displacement results indicate that fluorescence enhancement is governed not only by ligand binding, but also by the specific protein-bound conformation populated following HSA recognition and subsequent allosteric perturbation. Similar conformational-selection and allosteric mechanisms have been recognized as fundamental principles governing protein–ligand recognition in diverse biological systems [33,34,35].

The protein selectivity of sensors **1a**–**1f** was evaluated using bovine serum albumin (BSA), lysozyme, fibrinogen, and trypsin (Figure 5 and Figure S27). All six sensors exhibited pronounced fluorescence enhancement in the presence of BSA, with **1d** producing the largest response of approximately 40-fold. Although the probes did not consistently discriminate between HSA and BSA, their strong responses to both proteins indicate preferential recognition of serum albumins compared to other proteins. This behavior is consistent with the conserved hydrophobic binding environments of HSA and BSA. The conformational flexibility of the diene backbone may facilitate recognition of both proteins by allowing the sensors to adopt different *cisoid* and *transoid* geometries within their respective binding pockets. In contrast, the other proteins tested produced little fluorescence enhancement, while fibrinogen and human gamma globulin elicited only modest responses.

### 3.4. Molecular Docking

Molecular docking studies were performed using AutoDock 4.2.6 to determine the binding locations of the chalcone probes within HSA. A global docking analysis was performed rather than restricting the search to Drug Site III to ensure that the observed hemin displacement resulted from direct competition for a common binding site rather than long-range allosteric effects, which are known to occur in HSA [36]. The docking results predicted that all six chalcones bind within the hemin-binding site (Drug Site III), consistent with the fluorescence displacement studies. Notably, the two lowest-energy docking poses for every derivative were localized within Drug Site III, whereas only selected higher-energy poses occupied alternative binding locations (Tables S2–S7). The docked pose of chalcone **1a** was superimposed with that of hemin as a representative member of the chalcone series (Figure 6) showing the two ligands exhibit substantial spatial overlap within the hemin-binding pocket. Across the series, the chalcones interacted with a conserved set of residues lining Drug Site III, most frequently Arg117, Lys137, Phe134, Met123, Tyr138, Tyr161, Leu115, and Arg186 (Tables S2–S7), although derivative-dependent methoxy substitution introduced subtle differences in the predicted polar interactions without altering the preferred binding site.

The docking studies also revealed a distinct conformational preference of these sensors upon protein binding. The lowest-energy binding pose adopted by every derivative except **1c** adopted an *s*-cisoid conformation of the conjugated diene backbone, despite the transoid geometry being energetically favored in solution. A comparable HSA bound *s*-cisoid geometry was previously predicted for a structurally related diene-extended D-π-A probe suggesting that protein-induced conformational selection may be a recurring feature of HSA recognition by this class of sensors [37].

Furthermore, **1a**, **1b**, **1d**, **1e**, and **1f** retained the *s*-cisoid geometry across all three reported docking poses, while **1c** remained *s*-transoid in its two lowest-energy poses before adopting an *s*-cisoid geometry in the third pose (Tables S2–S7 and Figures S28–S33). Inspection of the individual docking conformations further revealed that, although the lowest-energy poses generally provided the most favorable predicted protein–ligand interactions, they frequently retained appreciable torsion along the conjugated backbone. In contrast, the second-ranked poses of several derivatives exhibited substantially greater backbone planarity while remaining energetically similar to the corresponding lowest-energy conformations (Tables S2–S7 and Figures S28–S33). For **1b**–**1f**, the two lowest-energy poses differed by only 0.12–0.50 kcal mol^−1^, suggesting that multiple bound conformations may be thermally accessible within the hemin-binding pocket. While the lowest-energy pose likely represents the most favorable protein–ligand interaction, the more planar conformations are expected to better preserve electronic conjugation, thereby enhancing intramolecular charge transfer while reducing torsional nonradiative relaxation. Consequently, the observed fluorescence response may arise from the relative populations and photophysical efficiencies of multiple closely spaced, bound conformational states rather than exclusively from the single lowest-energy docking pose. These findings suggest that the conformational flexibility of the diene linker enables HSA to bias the bound conformational ensemble toward *s*-cisoid geometries that maximize geometric complementarity while favoring the highly emissive states responsible for fluorescence activation.

## 4. Conclusions

Six diene-extended chalcones (**1a**–**1f**) were established as red-emissive HSA sensors with limits of detection ranging from 11 to 160 nM. Their fluorescence turn-on results from conversion of weakly emissive aggregate-like populations in PBS into emissive HSA-bound ensembles. Systematic methoxy substitution modulated binding behavior and fluorescence output without altering this general sensing mechanism, demonstrating the structural tunability of the scaffold.

This work identified a conformational and allosterically responsive sensing mechanism at the noncanonical FA1/hemin binding site. Hemin displacement and molecular docking supported localization of the sensors within FA1. Modeling generally predicted an *s*-cisoid lowest-energy binding orientation, with **1c** representing the notable transoid exception, together with energetically accessible alternative conformations differing in planarity. Moreover, ligands occupying distal Sudlow sites produced substituent-dependent fluorescence changes, with **1c** exhibiting the largest allosterically induced enhancement following ibuprofen binding. These results support a model in which distal ligand binding redistributes protein-bound sensor conformations and thereby modulates emission. Collectively, this work establishes diene-extended chalcones as sensitive, tunable HSA probes and identifies control of protein-bound conformational ensembles as a design strategy for FA1 targeted fluorescent sensors.

## Figures and Tables

**Scheme 1:**
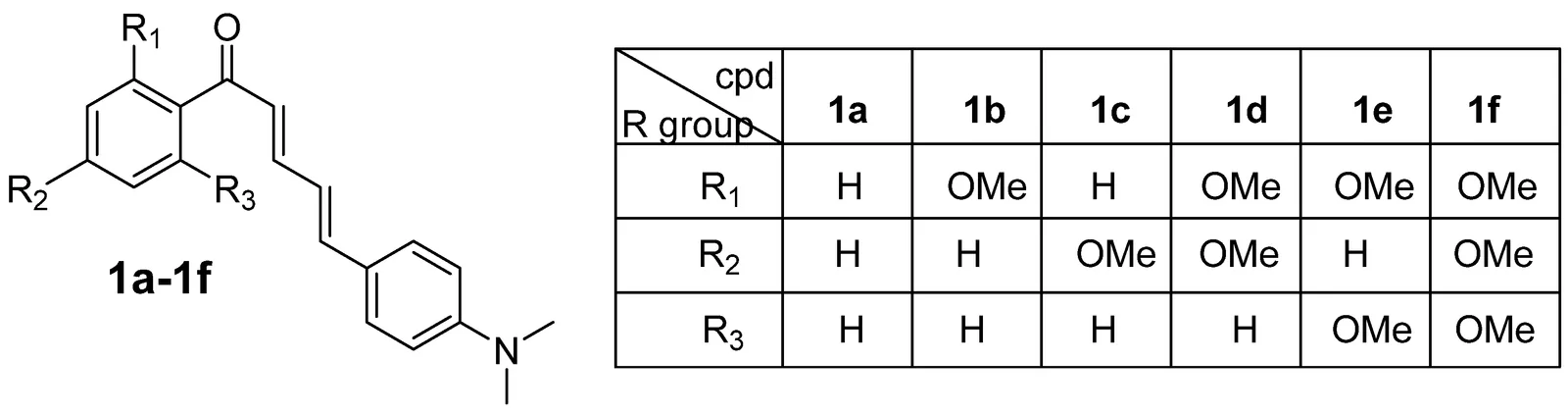
Structures of chalcone probes **1a**–**1f**.

**Figure 1:**
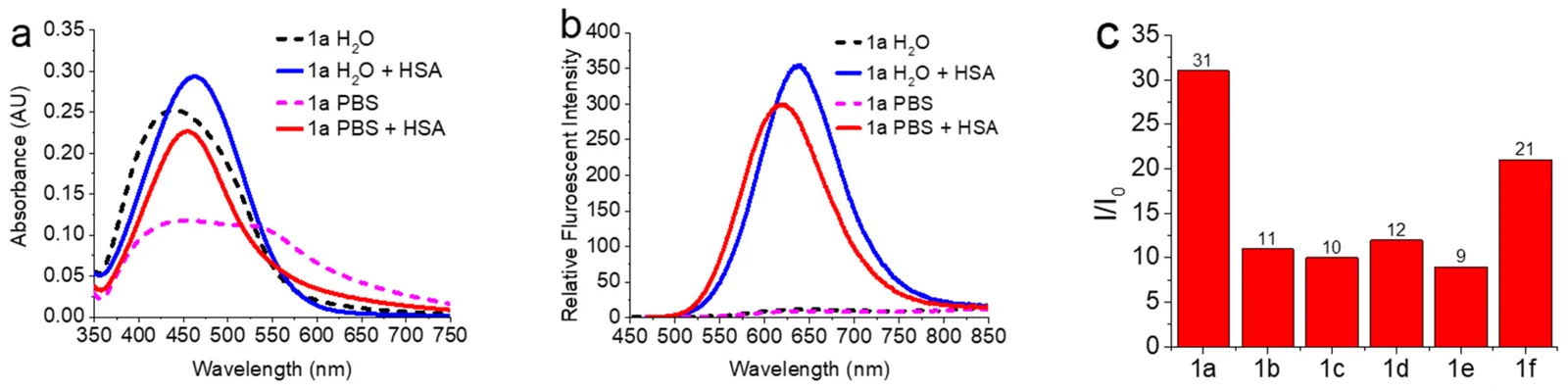
(**a**) Absorbance response of **1a** (10 µM) to HSA (18.3 µM) in water and PBS. (**b**) Fluorescent response of **1a** (10 µM) to HSA (18.3 µM) in water and PBS. (**c**) Fluorescence enhancement (*I*/*I*_0_) of chalcones **1a**–**1f** upon addition of HSA in PBS, where *I*_0_ and *I* represent the fluorescence intensities at the emission maximum in the absence and presence of HSA, respectively.

**Figure 2:**
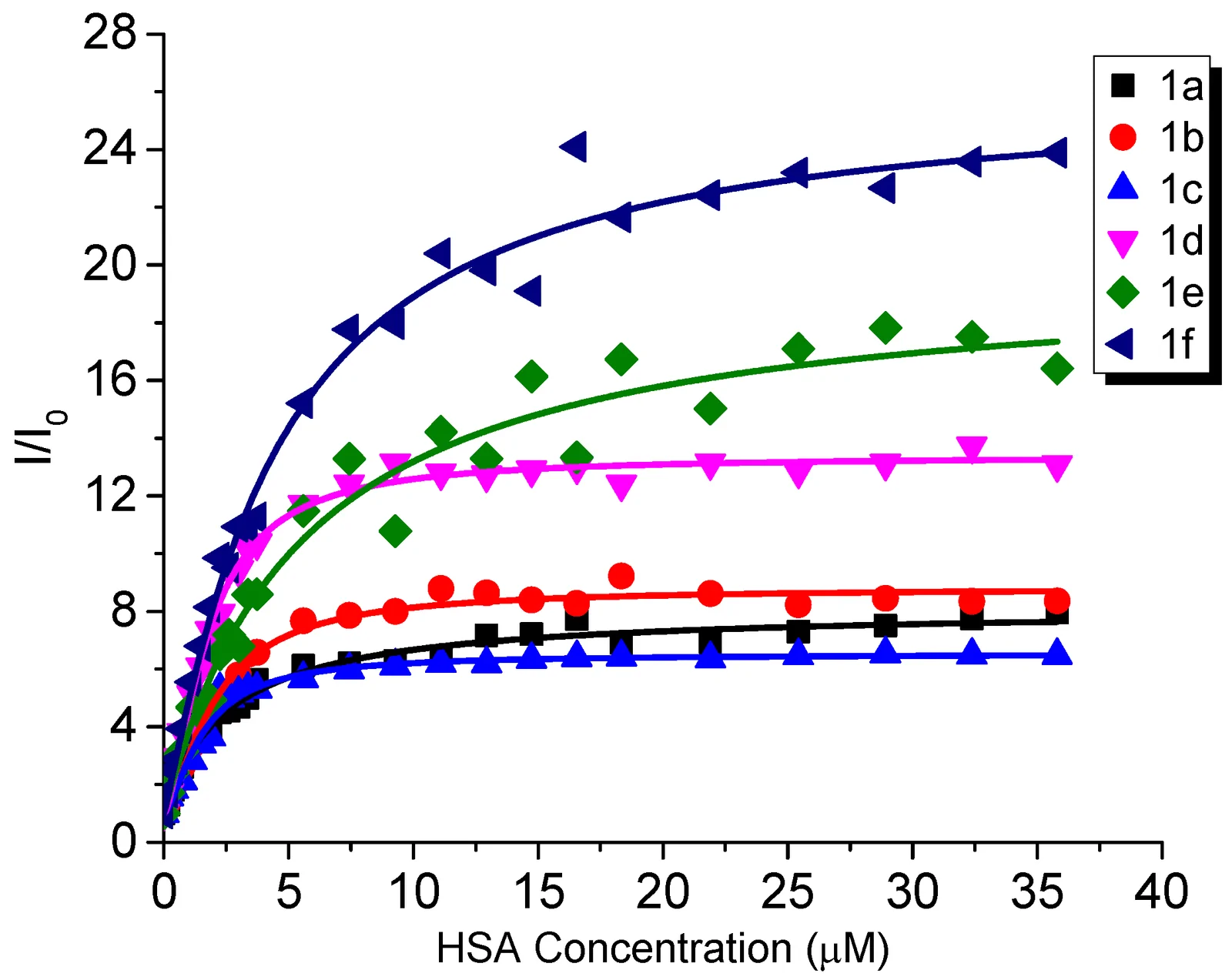
Fluorescence titration profiles of chalcones **1a**–**1f** upon incremental addition of HSA (18.3 µM) in PBS. The fluorescence response is expressed as $I/I_{0}$, where I and $I_{0}$ represent the integrated fluorescence intensities in the presence and absence of HSA, respectively. Solid lines represent fits to the Hill equation used to estimate the apparent binding parameters.

**Figure 3:**
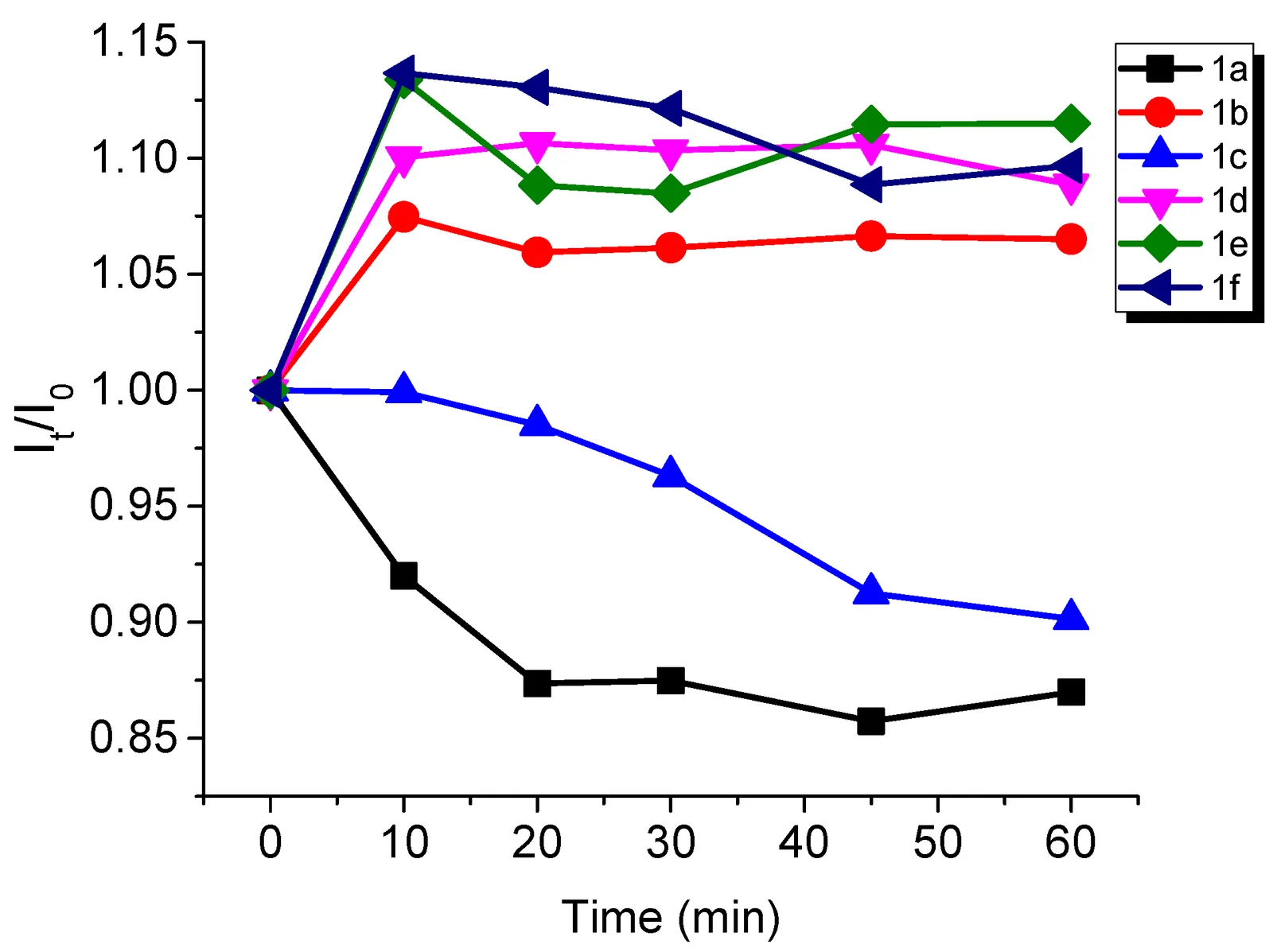
Time-dependent evolution of the fluorescence response of HSA-bound chalcones **1a**–**1f** (10 µM) in PBS. The fluorescence response is expressed as $I_{t}/I_{0}$, where $I_{t}$ is the integrated fluorescence intensity at time t, and $I_{0}$ is the integrated fluorescence intensity immediately following addition of HSA (35.8 µM).

**Figure 4:**
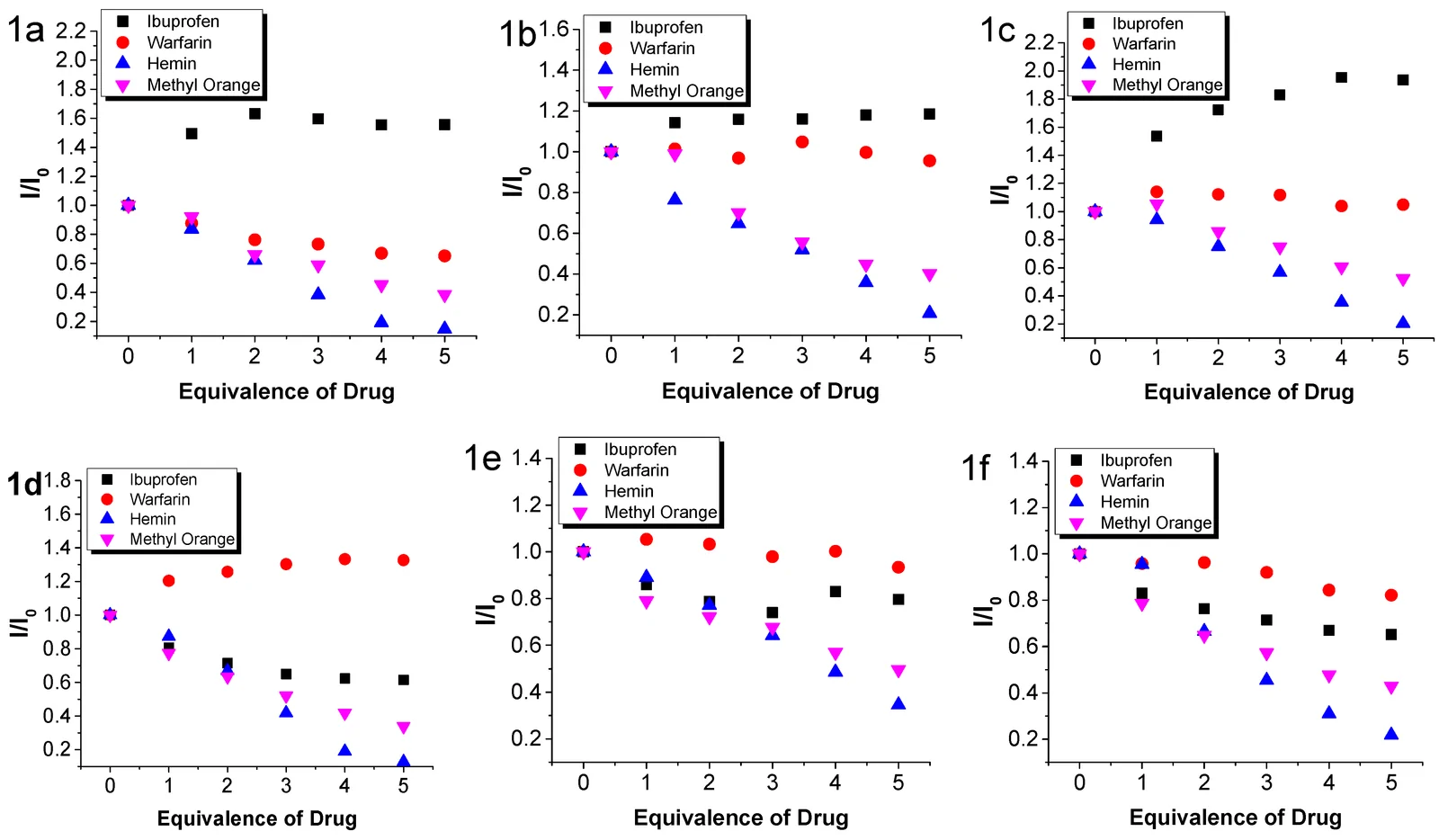
Competitive binding studies of chalcones **1a**–**1f** (10 µM) in the presence of HSA (18.3 µM). Changes in integrated fluorescence intensity ($I/I_{0}$) of the HSA-bound probe upon sequential addition of ibuprofen, warfarin, hemin, or methyl orange (0–5 equiv., relative to the chalcone concentration). $I_{0}$ represents the integrated fluorescence intensity of the HSA-bound probe prior to addition of the competing ligand.

**Figure 5:**
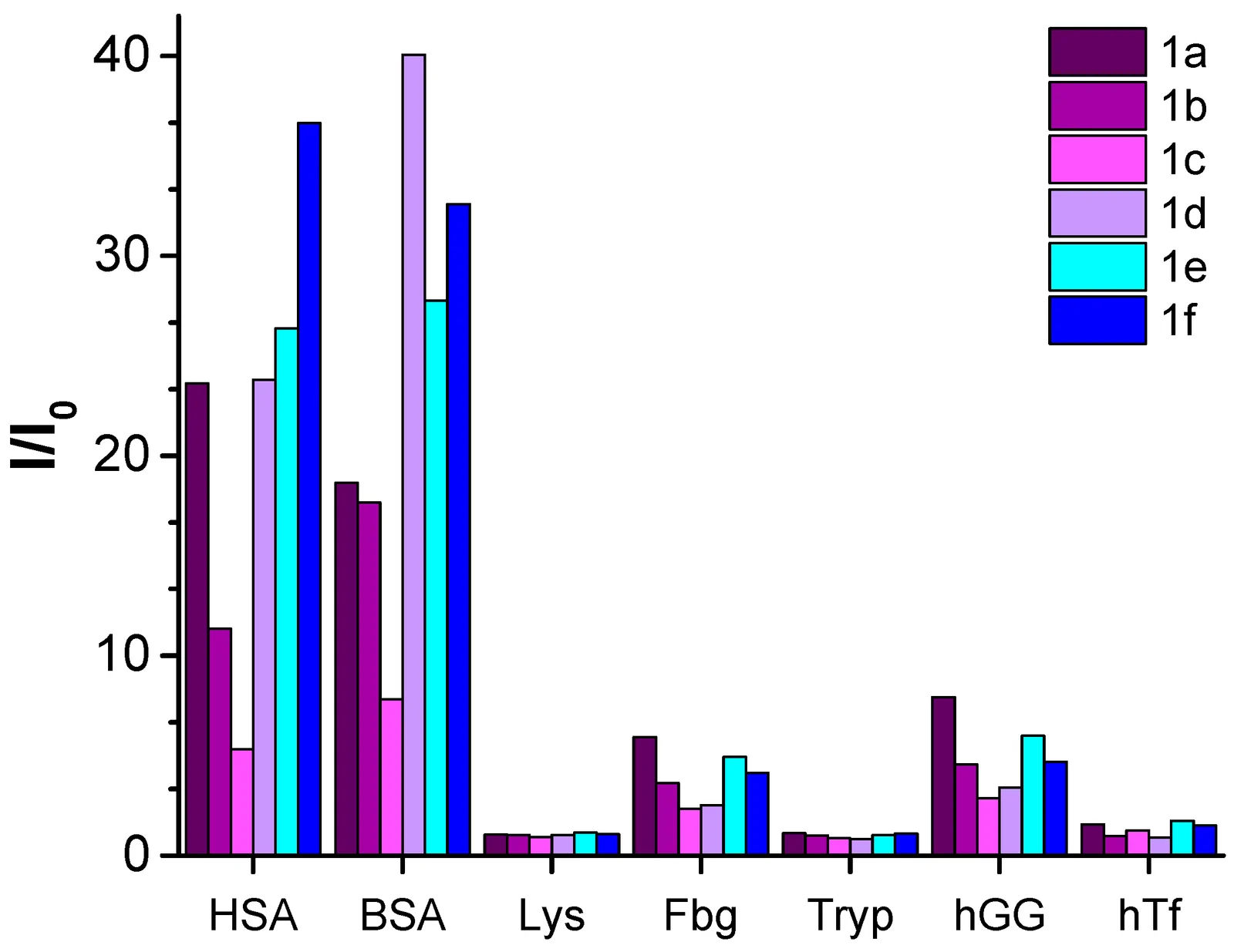
Fluorescent intensity changes (*I/I*_0_) of chalcone sensors **1a**–**1f** (2.5 μM) in the presence of HSA, BSA, lysozyme (Lys), fibrinogen (Fbg), trypsin (Tryp), human gamma globulin (hGG), and human transferrin (hTf) at 5 μM in PBS (pH 7.4). *I* and *I*_0_ represent the integrated fluorescence intensities in the presence and absence of protein, respectively.

**Figure 6:**
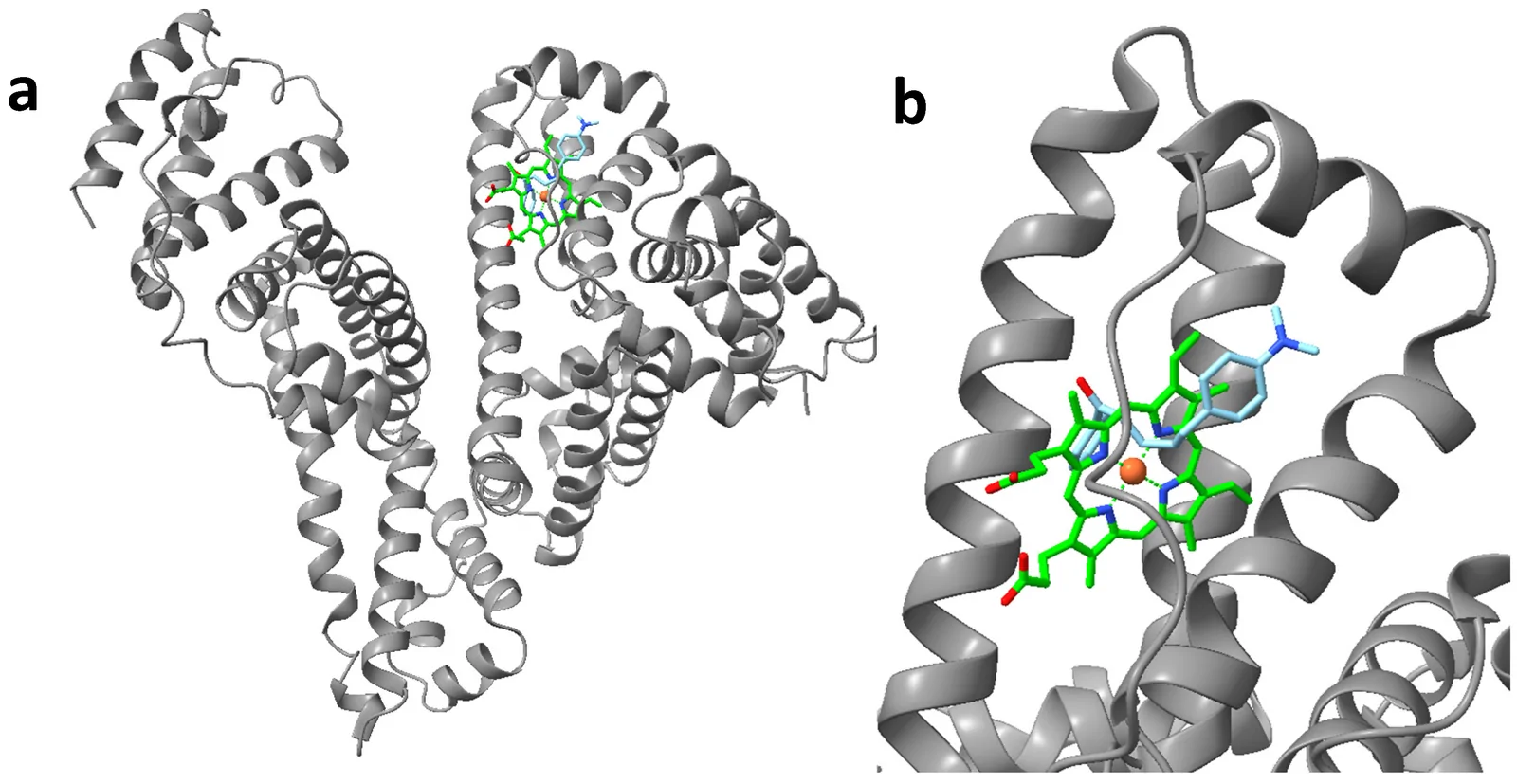
Structural overlay of the docked poses of chalcone **1a** and hemin within human serum albumin (HSA). (**a**) Global view of HSA showing the superimposed docked poses of chalcone **1a** (cyan) and hemin (green). (**b**) Enlarged view of the hemin-binding pocket showing the overlapping binding poses of the two ligands. The N, O and Fe atoms in the molecules are shown in blue, red and orange colors, respectively.

**Table 1:** Binding parameters and analytical performance metrics obtained from fluorescence titrations of chalcones **1a**–**1f** with HSA in PBS.

Sensor	Apparent K (µM)	Hill *n*	Reduced Χ^2^	LOD (nM)	LOQ (nM)	R^2^
**1a**	2.54	1.00	0.074	24	79	0.987
**1b**	2.04	1.48	0.085	11	36	0.992
**1c**	1.55	1.51	0.064	101	335	0.997
**1d**	1.77	1.55	0.091	160	535	0.998
**1e**	5.44	1.02	0.913	23	78	0.993
**1f**	4.45	1.13	0.677	23	78	0.997

Apparent K: apparent binding affinity to HSA; Hill *n*: Hill coefficient.

## Data Availability

Data available upon request.

## Supplementary Materials

The following supporting information can be downloaded at https://www.mdpi.com/article/10.3390/bios16100533/s1, Figure S1: Absorbance (dotted lines) and fluorescence (solid lines) of chalcones **1a**–**1f** (10 µM) in the presence of HSA (18.3 µM); Figure S2: Concentration dependent absorption spectra of sensors **1a**–**1f** in PBS (0.625–12.5 µM) recorded in the absense of HSA to evaluate concentration-dependent aggregation; Figure S3: Linear regression plot used for determination of the limits of detection (LOD) and quantification (LOQ) for chalcone **1a** toward HSA in PBS. The blank responses used to determine σ are shown to the right where Int. Fl indicates integrated fluorescence (CPS—counts per second) and STDV is the standard deviation; Figure S4: Linear regression plot used for determination of the limits of detection (LOD) and quantification (LOQ) for chalcone **1b** toward HSA in PBS. The blank responses used to determine σ are shown to the right where Int. Fl indicates integrated fluorescence (CPS—counts per second) and STDV is the standard deviation; Figure S5: Linear regression plot used for determination of the limits of detection (LOD) and quantification (LOQ) for chalcone **1c** toward HSA in PBS. The blank responses used to determine σ are shown to the right where Int. Fl indicates integrated fluorescence (CPS—counts per second) and STDV is the standard deviation; Figure S6. Linear regression plot used for determination of the limits of detection (LOD) and quantification (LOQ) for chalcone **1d** toward HSA in PBS. The blank responses used to determine σ are shown to the right where Int. Fl indicates integrated fluorescence (CPS—counts per second) and STDV is the standard deviation; Figure S7: Linear regression plot used for determination of the limits of detection (LOD) and quantification (LOQ) for chalcone **1e** toward HSA in PBS. The blank responses used to determine σ are shown to the right where Int. Fl indicates integrated fluorescence (CPS—counts per second) and STDV is the standard deviation; Figure S8: Linear regression plot used for determination of the limits of detection (LOD) and quantification (LOQ) for chalcone **1f** toward HSA in PBS. The blank responses used to determine σ are shown to the right where Int. Fl indicates integrated fluorescence (CPS—counts per second) and STDV is the standard deviation; Figure S9: Absorption of sensor **1a** acquired during titration with 5% HSA in PBS; Figure S10: Absorbance of sensor **1b** acquired during titration with 5% HSA in PBS; Figure S11: Absorbance of sensor **1c** acquired during titration with 5% HSA in PBS; Figure S12: Absorbance of sensor **1d** acquired during titration with 5% HSA in PBS; Figure S13: Absorbance of sensor **1e** acquired during titration with 5% HSA in PBS; Figure S14: Absorbance of sensor **1f** acquired during titration with 5% HSA in PBS; Figure S15: Fluorescent response of sensor **1a** acquired during titration with 5% HSA in PBS; Figure S16: Fluorescent response of sensor **1b** acquired during titration with 5% HSA in PBS; Figure S17: Fluorescent response of sensor **1c** acquired during titration with 5% HSA in PBS; Figure S18: Fluorescent response of sensor **1d** acquired during titration with 5% HSA in PBS; Figure S19: Fluorescent response of sensor **1e** acquired during titration with 5% HSA in PBS; Figure S20: Fluorescent response of sensor **1f** acquired during titration with 5% HSA in PBS; Figure S21: Time-dependent absorption (a) and fluorescence (b) spectra of chalcone **1a** in PBS following addition of HSA (35.8 µM). Spectra were recorded over a 60 min period to monitor the stability and evolution of the protein-bound state; Figure S22: Time-dependent absorption (a) and fluorescence (b) spectra of chalcone **1b** in PBS following addition of HSA (35.8 µM). Spectra were recorded over a 60 min period to monitor the stability and evolution of the protein-bound state; Figure S23: Time-dependent absorption (a) and fluorescence (b) spectra of chalcone **1c** in PBS following addition of HSA (35.8 µM). Spectra were recorded over a 60 min period to monitor the stability and evolution of the protein-bound state; Figure S24: Time-dependent absorption (a) and fluorescence (b) spectra of chalcone **1d** in PBS following addition of HSA (35.8 µM). Spectra were recorded over a 60 min period to monitor the stability and evolution of the protein-bound state; Figure S25: Time-dependent absorption (a) and fluorescence (b) spectra of chalcone **1e** in PBS following addition of HSA (35.8 µM). Spectra were recorded over a 60 min period to monitor the stability and evolution of the protein-bound state; Figure S26: Time-dependent absorption (a) and fluorescence (b) spectra of chalcone **1f** in PBS following addition of HSA (35.8 µM). Spectra were recorded over a 60 min period to monitor the stability and evolution of the protein-bound state; Figure S27: Fluorescence response of chalcone sensors **1a**–**1f** (2.5 μM) in the presence of HSA, BSA, lysozyme, fibrinogen, and trypsin (5 μM) in PBS (pH 7.4); Figure S28: Representative molecular docking poses of chalcone **1a** within the HSA hemin-binding pocket (FA1). Panels (**a**,**b**) show the lowest-energy docking pose (Pose 1), panels (**c**,**d**) show the second-ranked docking pose (Pose 2). For each pose, the left panel depicts the ligand within the protein surface, while the right panel highlights the surrounding binding pocket residues. Corresponding binding energies are provided in Table S2; Figure S29: Representative molecular docking poses of chalcone **1b** within the HSA hemin-binding pocket (FA1). Panels (**a**,**b**) show the lowest-energy docking pose (Pose 1), panels (**c**,**d**) show the second-ranked docking pose (Pose 2), and panels (**e**,**f**) show the third-ranked docking pose (Pose 3). For each pose, the left panel depicts the ligand within the protein surface, while the right panel highlights the surrounding binding pocket residues. Corresponding binding energies are provided in Table S3; Figure S30: Representative molecular docking poses of chalcone **1c** within the HSA hemin-binding pocket (FA1). Panels (**a**,**b**) show the lowest-energy docking pose (Pose 1), panels (**c**,**d**) show the second-ranked docking pose (Pose 2), and panels (**e**,**f**) show the third-ranked docking pose (Pose 3). For each pose, the left panel depicts the ligand within the protein surface, while the right panel highlights the surrounding binding pocket residues. Corresponding binding energies are provided in Table S4; Figure S31: Representative molecular docking poses of chalcone **1d** within the HSA hemin-binding pocket (FA1). Panels (**a**,**b**) show the lowest-energy docking pose (Pose 1), panels (**c**,**d**) show the second-ranked docking pose (Pose 2), and panels (**e**,**f**) show the third-ranked docking pose (Pose 3). For each pose, the left panel depicts the ligand within the protein surface, while the right panel highlights the surrounding binding pocket residues. Corresponding binding energies are provided in Table S5; Figure S32: Representative molecular docking poses of chalcone **1e** within the HSA hemin-binding pocket (FA1). Panels (**a**,**b**) show the lowest-energy docking pose (Pose 1), panels (**c**,**d**) show the second-ranked docking pose (Pose 2). For each pose, the left panel depicts the ligand within the protein surface, while the right panel highlights the surrounding binding pocket residues. Corresponding binding energies are provided in Table S6; Figure S33: Representative molecular docking poses of chalcone **1f** within the HSA hemin-binding pocket (FA1). Panels (**a**,**b**) show the lowest-energy docking pose (Pose 1), panels (**c**,**d**) show the second-ranked docking pose (Pose 2). For each pose, the left panel depicts the ligand within the protein surface, while the right panel highlights the surrounding binding pocket residues. Corresponding binding energies are provided in Table S7; Table S1: Absorbance and fluorescence wavelength maxima of chalcones **1a**–**1f** (10µM) in the presence of HSA (18.3 µM); Table S2: Docking parameters and residue interactions for chalcone **1a** across the three lowest energy binding poses. Values include binding energy, ligand efficiency, inhibition constant, geometry, contact distances, residues in close contact, and polar interactions; Table S3: Docking parameters and residue interactions for chalcone **1b** across the three lowest energy binding poses. Values include binding energy, ligand efficiency, inhibition constant, geometry, contact distances, residues in close contact, and polar interactions; Table S4: Docking parameters and residue interactions for chalcone **1c** across the three lowest energy binding poses. Values include binding energy, ligand efficiency, inhibition constant, geometry, contact distances, residues in close contact, and polar interactions; Table S5: Docking parameters and residue interactions for chalcone **1d** across the three lowest energy binding poses. Values include binding energy, ligand efficiency, inhibition constant, geometry, contact distances, residues in close contact, and polar interactions; Table S6: Docking parameters and residue interactions for chalcone **1e** across the three lowest energy binding poses. Values include binding energy, ligand efficiency, inhibition constant, geometry, contact distances, residues in close contact, and polar interactions; Table S7: Docking parameters and residue interactions for chalcone **1f** across the three lowest energy binding poses. Values include binding energy, ligand efficiency, inhibition constant, geometry, contact distances, residues in close contact, and polar interactions.
