# Supplementary material for: Diene-Extended Chalcones Enable Red Fluorescence Detection of Human Serum Albumin Through FA1 Binding
Source: Biosensors (Basel). 2026 Sep 24;16(10):533. doi: 10.3390/bios16100533 (PMC13702613; doi:10.3390/bios16100533)
Supplement: Supplementary file 1 [file biosensors-16-00533-s001.zip › biosensors-4520207-supplementary.pdf]

biosensors

Article

Diene-Extended Chalcones Enable Red Fluorescence Detection of Human Serum Albumin through FA1 Binding

Brian Corbin , Jessica Mason , Paityn Houglan , Amanda Murphey and Yi Pang *

Department of Chemistry, The University of Akron, Akron, OH 44325, USA; bpc32@uakron.edu(B.C.); jlm479@uakron.edu(J.M.); pmhmed15@gmail.com(P.H.); am961@uakron.edu(A.M.)

* Correspondence: yp5@uakron.edu

1. Absorbance and fluorescence spectra of chalcones **1a–1f** in H2O and PBS with HSA (3-5)
2. Linear regression analyses for determination of LOD and LOQ (6-11)
3. Absorbance and fluorescence titrations of chalcones **1a–1f** with HSA (12-17)
4. Time-dependent absorbance and fluorescence responses of chalcones **1a–1f** following HSA binding (18-20)
5. Fluorescence response of **1a–1f** to various proteins (21)
6. Molecular docking parameters and predicted protein–ligand interactions (22-24)
7. Predicted binding conformations of chalcones **1a–1f** in the FA1 binding pocket (25-30)

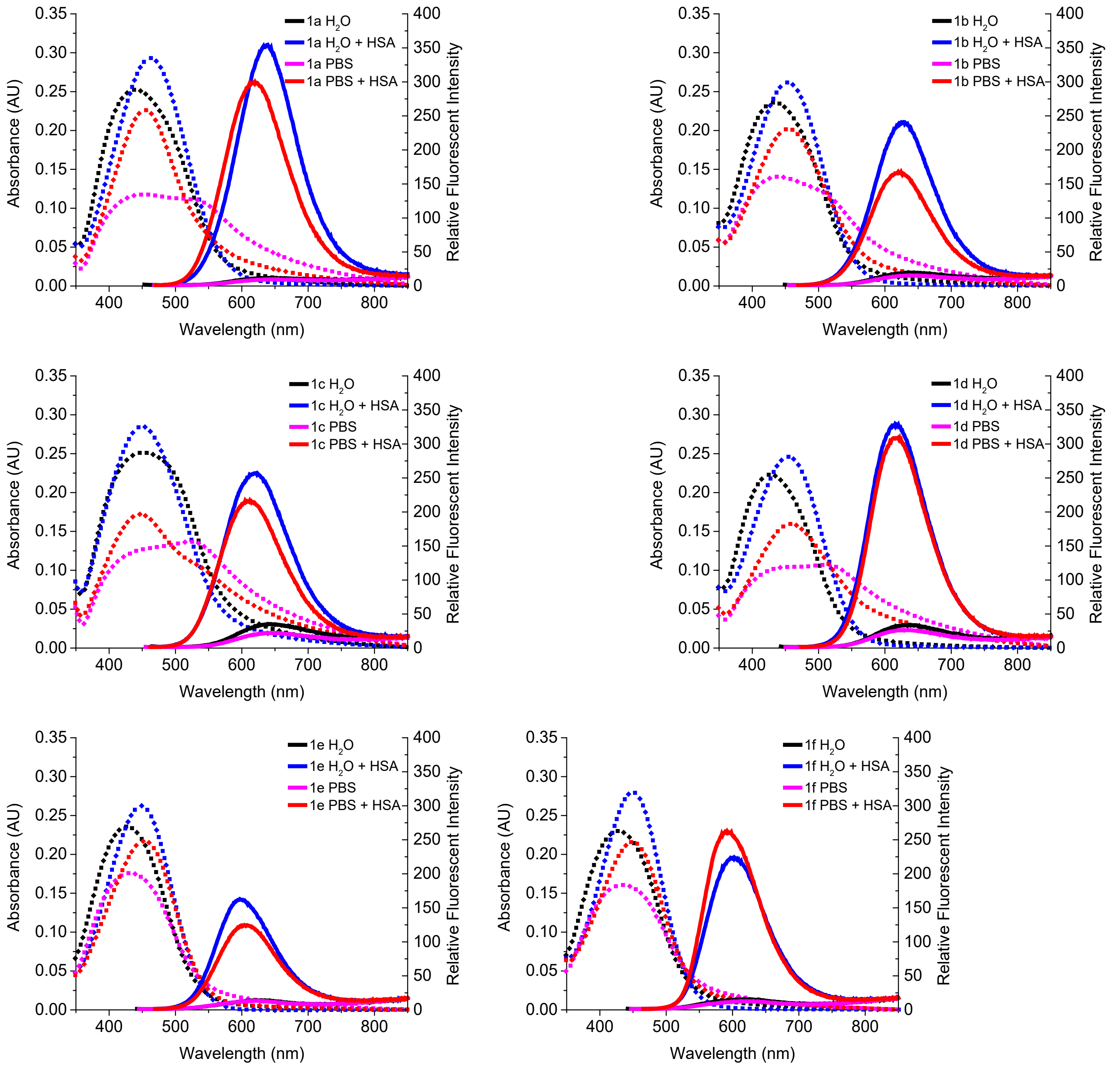

Figure S1. Absorbance (dotted lines) and fluorescence (solid lines) of chalcones **1a-1f** (10 μM) in the presence of HSA (18.3 μM).

Table S1. Absorbance and fluorescence wavelength maxima of chalcones **1a-1f** (10μM) in the presence of HSA (18.3 μM).

Compound	λ_{abs} H_2O (nm)	λ_{abs} PBS (nm)	λ_{em} H_2O (nm)	λ_{em} PBS (nm)	Fold enhancement (PBS)	Φ[1]
**1a**	439	446	641	648		
**1a + HSA**	463	454	638	619	31	0.129
**1b**	435	440	641	637		
**1b + HSA**	453	453	627	622	11	0.121
**1c**	454	440	643	643		
**1c + HSA**	451	447	619	612	10	0.080
**1d**	428	435	637	633		
**1d + HSA**	456	459	617	617	12	0.334
**1e**	426	429	626	620		
**1e + HSA**	449	453	598	605	9	0.115
**1f**	428	437	616	621		
**1f + HSA**	450	450	602	592	21	0.184

[1] Absolute quantum yields were acquired in PBS after incubating the senor with HSA (18.3 μM) for 30 minutes.

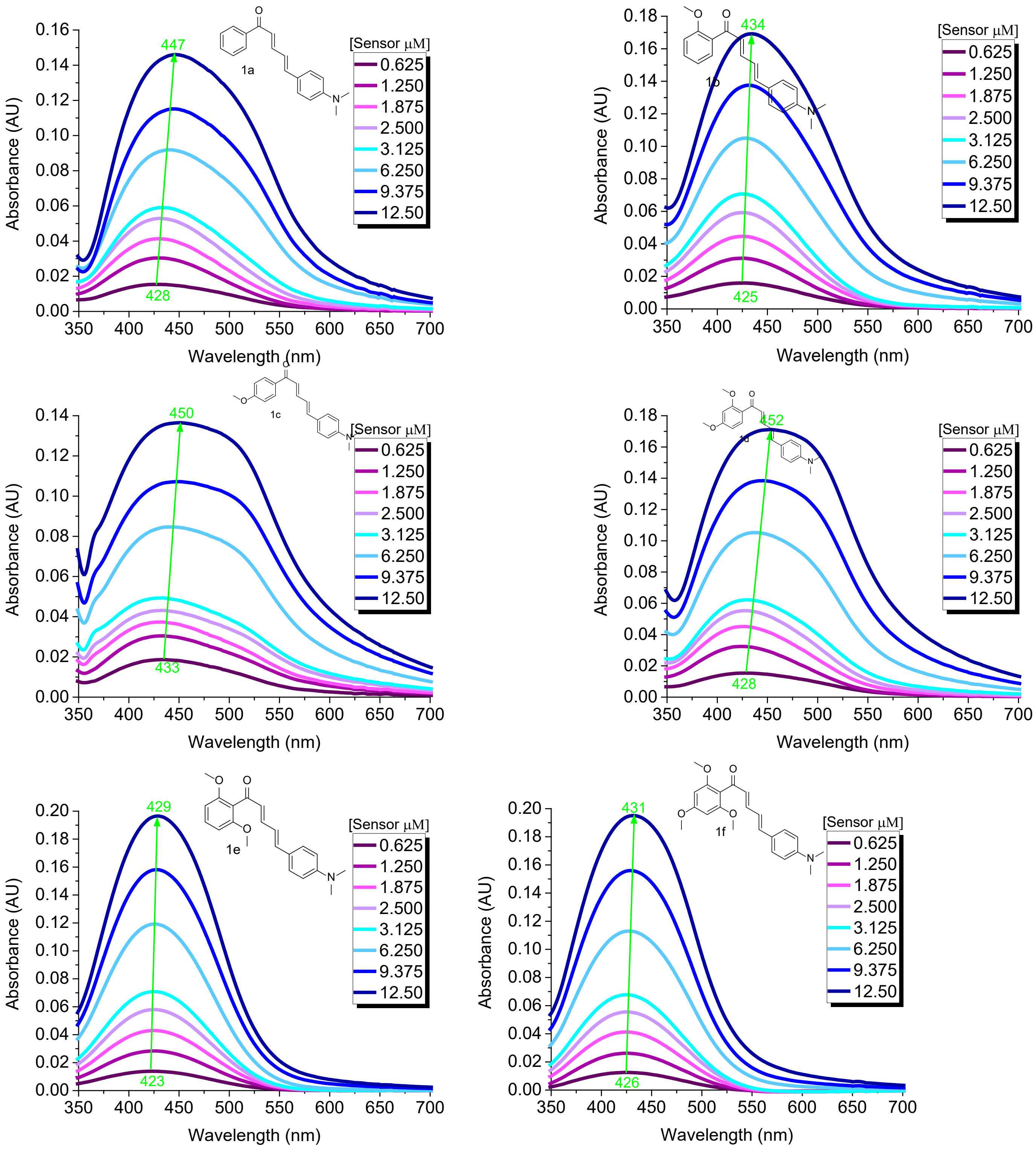

Figure S2. Concentration-dependent absorption spectra of sensors **1a–1f** in PBS (0.625–12.5 µM) recorded in the absense of HSA to evaluate concentration-dependent aggregation.

Limit of Detection (LOD) and Limit of Quantification (LOQ) were calculated from the linear region of the calibration curves using the 3σ/slope and 10σ/slope criteria, respectively, where σ represents the standard deviation of ten blank measurements (2.5 μM sensor in PBS). Integrated fluorescent intensity was plotted against HSA concentration to give the calibration curves below.

Example chalcone **1a (LOD)**:

σ = 322829 CPS

slope = 407076 * 10^7 CPS/μM

LOD = 3(322829 CPS)/(407076 * 10^7 CPS/μM)

LOD = 0.024 μM ≡ 24 nm

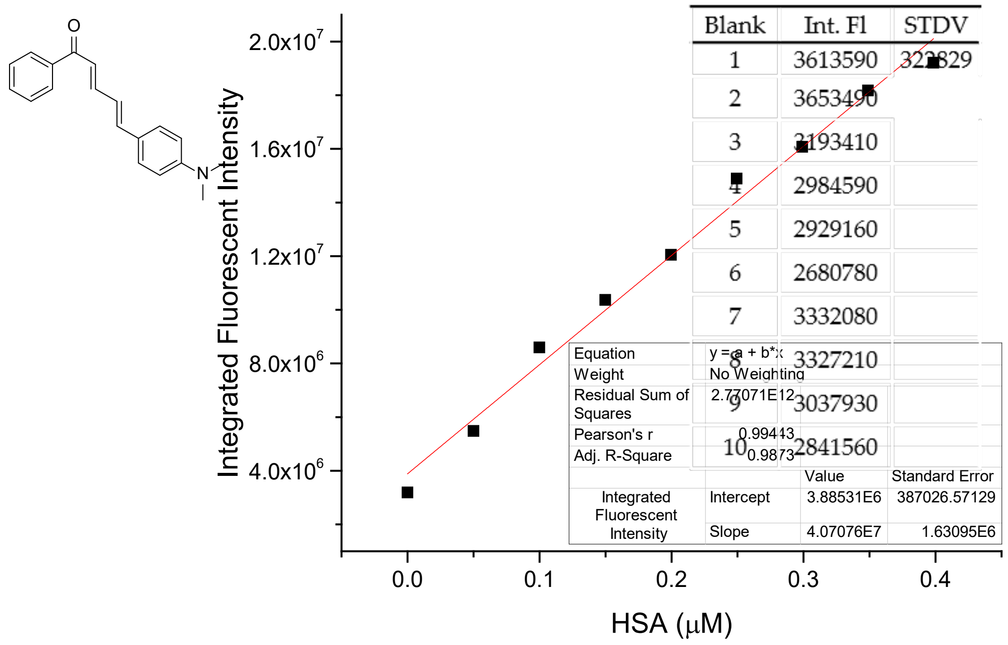

Blank	Int. Fl	STDV
1	3613590	322829
2	3653490	
3	3193410	
4	2984590	
5	2929160	
6	2680780	
7	3332080	
8	3327210	
9	3037930	
10	2841560	

Figure S3. Linear regression plot used for determination of the limits of detection (LOD) and quantification (LOQ) for chalcone **1a** toward HSA in PBS. The blank responses used to determine σ are shown to the right where Int. Fl indicates integrated fluorescence (CPS – counts per second) and STDV is the standard deviation.

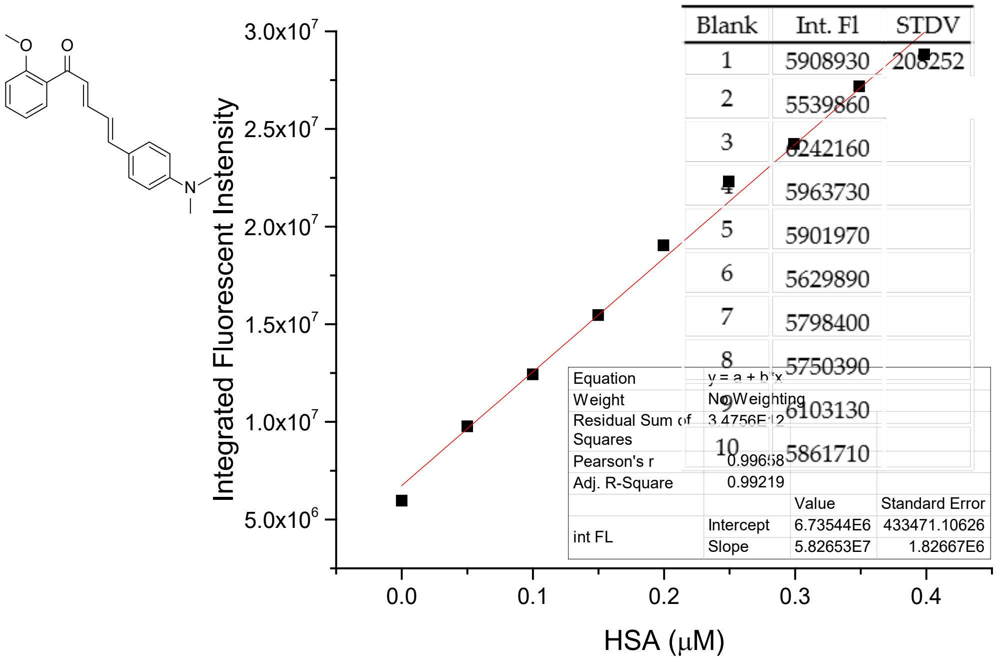

Figure S4. Linear regression plot used for determination of the limits of detection (LOD) and quantification (LOQ) for chalcone **1b** toward HSA in PBS. The blank responses used to determine σ are shown to the right where Int. Fl indicates integrated fluorescence (CPS – counts per second) and STDV is the standard deviation.

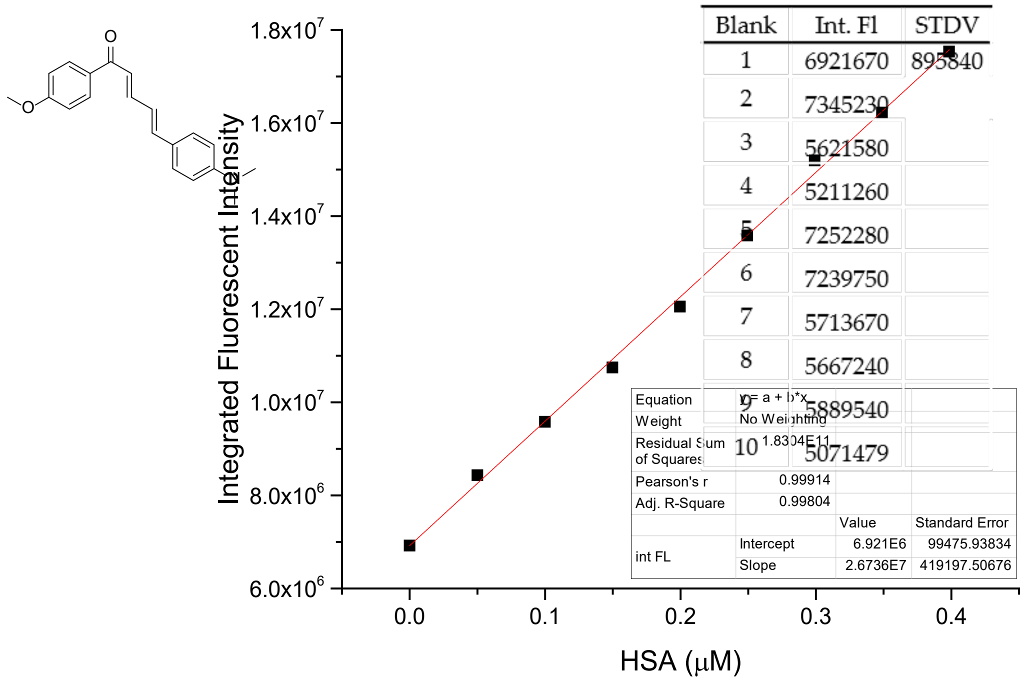

Blank	Int. Fl	STDV
1	6921670	895840
2	7345230	
3	5621580	
4	5211260	
5	7252280	
6	7239750	
7	5713670	
8	5667240	
9	5889540	
10	5071479	

Equation	y = a + b*x		
Weight	No Weighting		
Residual Sum of Squares	1.8304E11		
Pearson's r	0.99914		
Adj. R-Square	0.99804		
		Value	Standard Error
int FL	Intercept	6.921E6	99475.93834
	Slope	2.6736E7	419197.50676

Figure S5. Linear regression plot used for determination of the limits of detection (LOD) and quantification (LOQ) for chalcone **1c** toward HSA in PBS. The blank responses used to determine σ are shown to the right where Int. Fl indicates integrated fluorescence (CPS – counts per second) and STDV is the standard deviation.

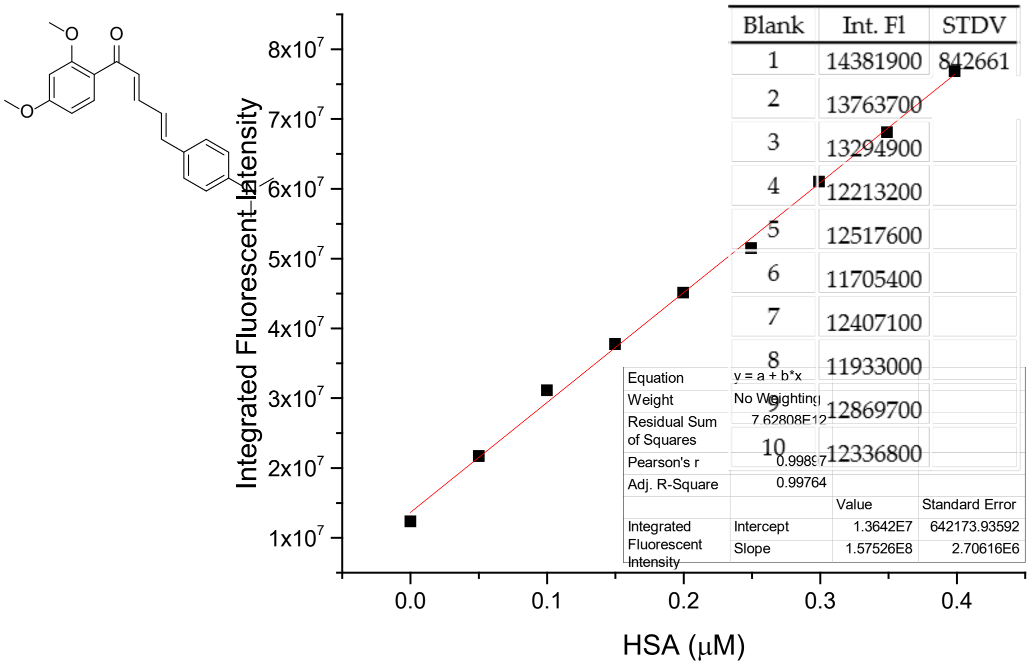

Figure S6. Linear regression plot used for determination of the limits of detection (LOD) and quantification (LOQ) for chalcone **1d** toward HSA in PBS. The blank responses used to determine σ are shown to the right where Int. Fl indicates integrated fluorescence (CPS – counts per second) and STDV is the standard deviation.

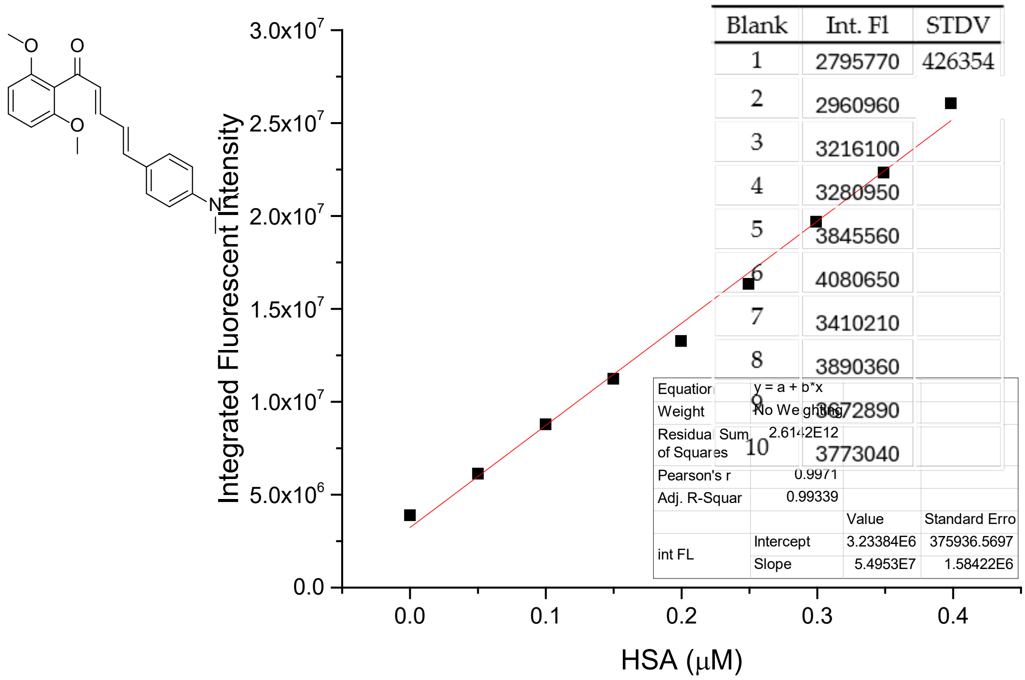

Figure S7. Linear regression plot used for determination of the limits of detection (LOD) and quantification (LOQ) for chalcone **1e** toward HSA in PBS. The blank responses used to determine σ are shown to the right where Int. Fl indicates integrated fluorescence (CPS – counts per second) and STDV is the standard deviation.

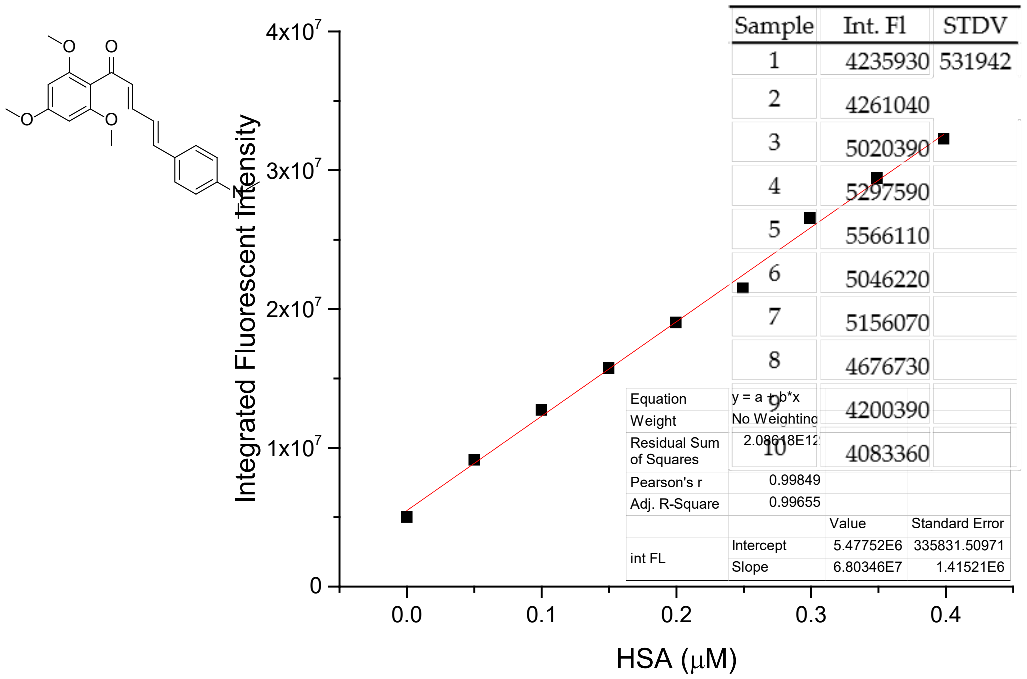

Sample	Int. Fl	STDV
1	4235930	531942
2	4261040	
3	5020390	
4	5297590	
5	5566110	
6	5046220	
7	5156070	
8	4676730	
9	4200390	
10	4083360	

Equation	y = a + b*x		
Weight	No Weighting		
Residual Sum of Squares	2.08618E12		
Pearson's r	0.99849		
Adj. R-Square	0.99655		
		Value	Standard Error
int FL	Intercept	5.47752E6	335831.50971
	Slope	6.80346E7	1.41521E6

Figure S8. Linear regression plot used for determination of the limits of detection (LOD) and quantification (LOQ) for chalcone **1f** toward HSA in PBS. The blank responses used to determine σ are shown to the right where Int. Fl indicates integrated fluorescence (CPS – counts per second) and STDV is the standard deviation.

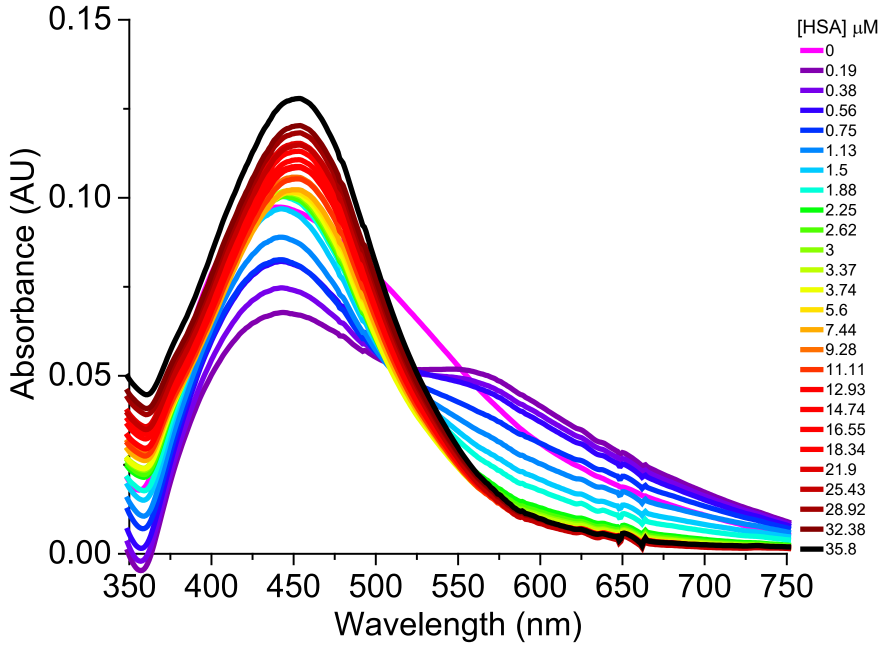

Figure S9. Absorption of sensor **1a** acquired during titration with 5% HSA in PBS.

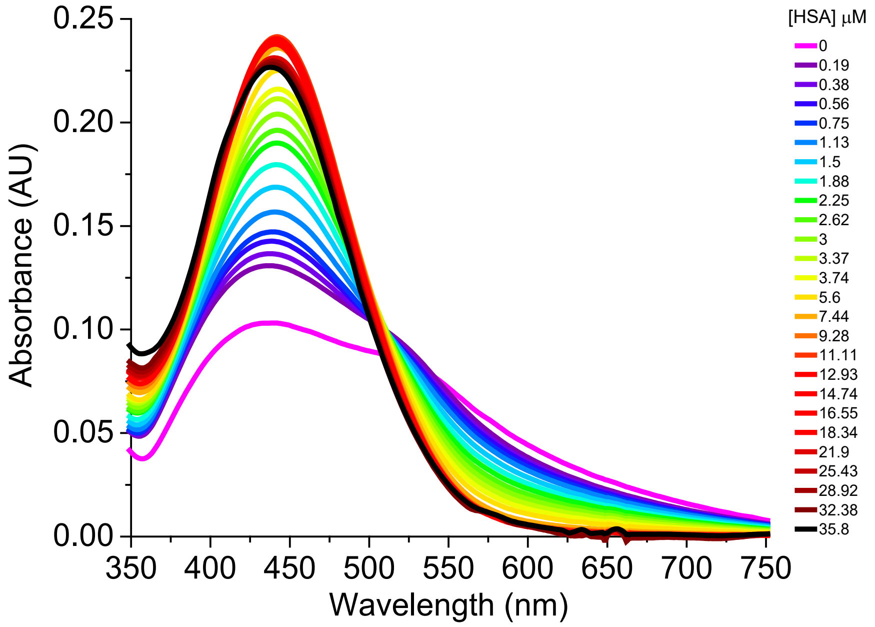

Figure S10. Absorbance of sensor **1b** acquired during titration with 5% HSA in PBS.

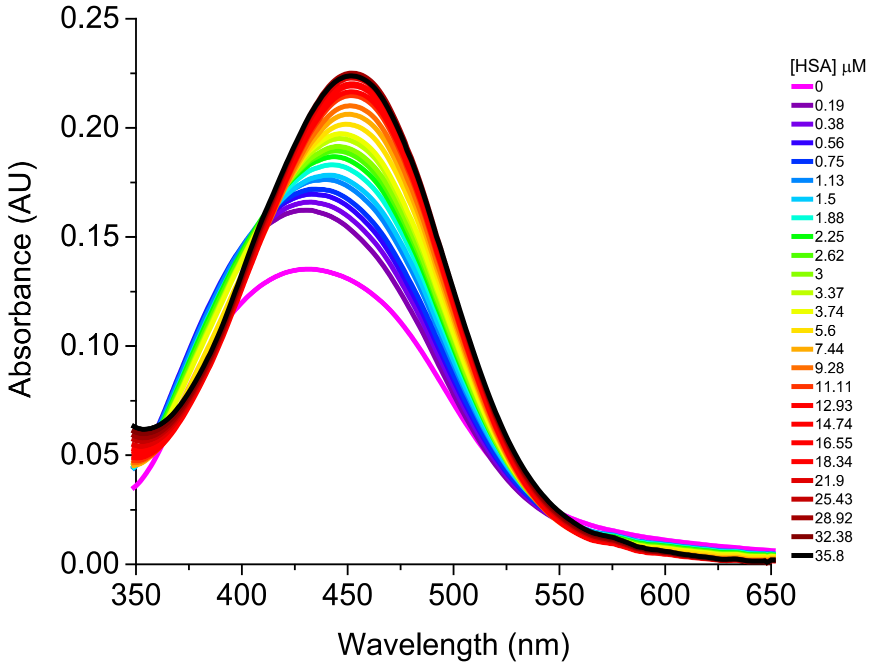

Figure S11. Absorbance of sensor **1c** acquired during titration with 5% HSA in PBS.

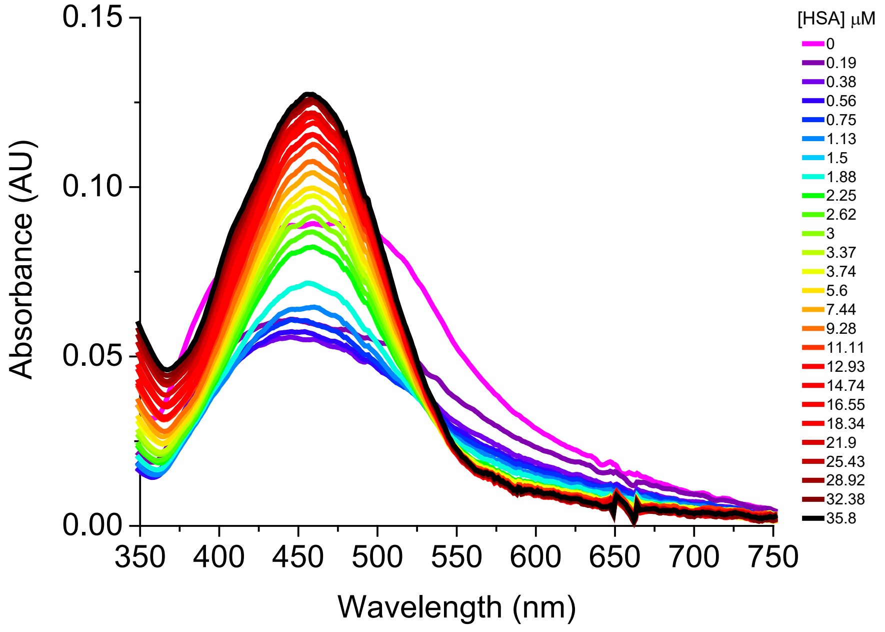

Figure S12. Absorbance of sensor **1d** acquired during titration with 5% HSA in PBS.

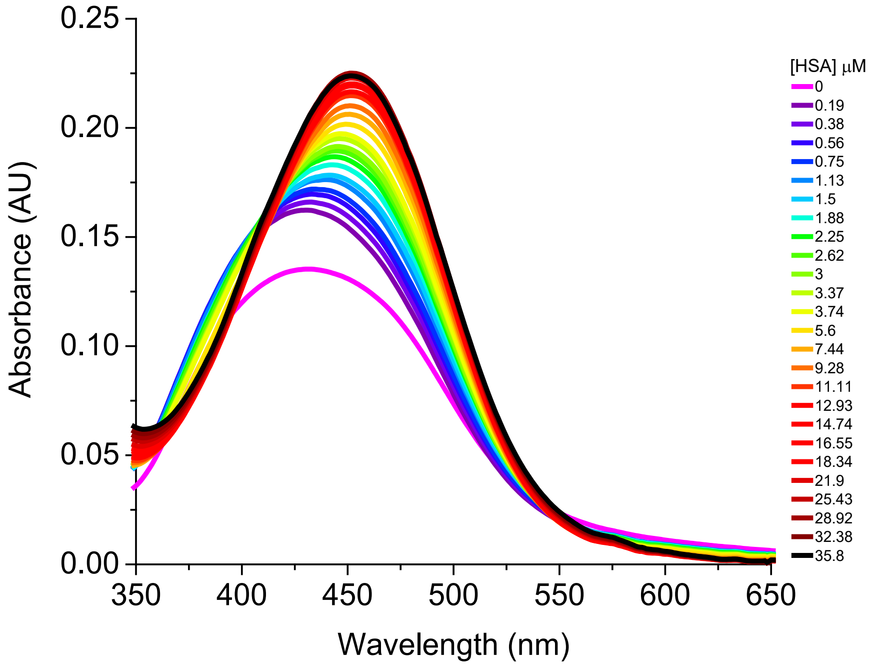

Figure S13. Absorbance of sensor **1e** acquired during titration with 5% HSA in PBS.

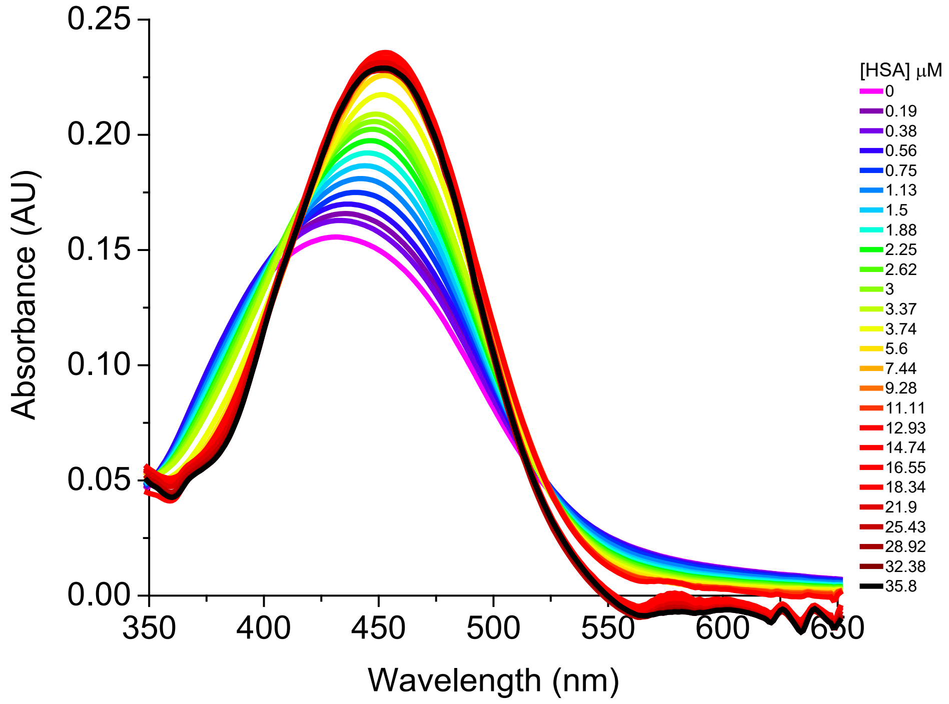

Figure S14. Absorbance of sensor **1f** acquired during titration with 5% HSA in PBS.

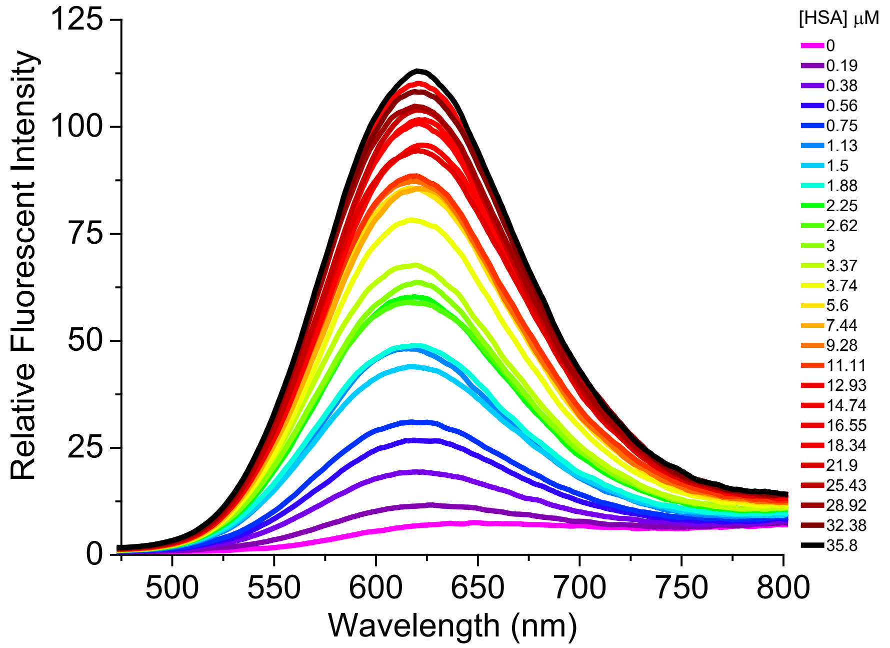

Figure S15. Fluorescent response of sensor **1a** acquired during titration with 5% HSA in PBS.

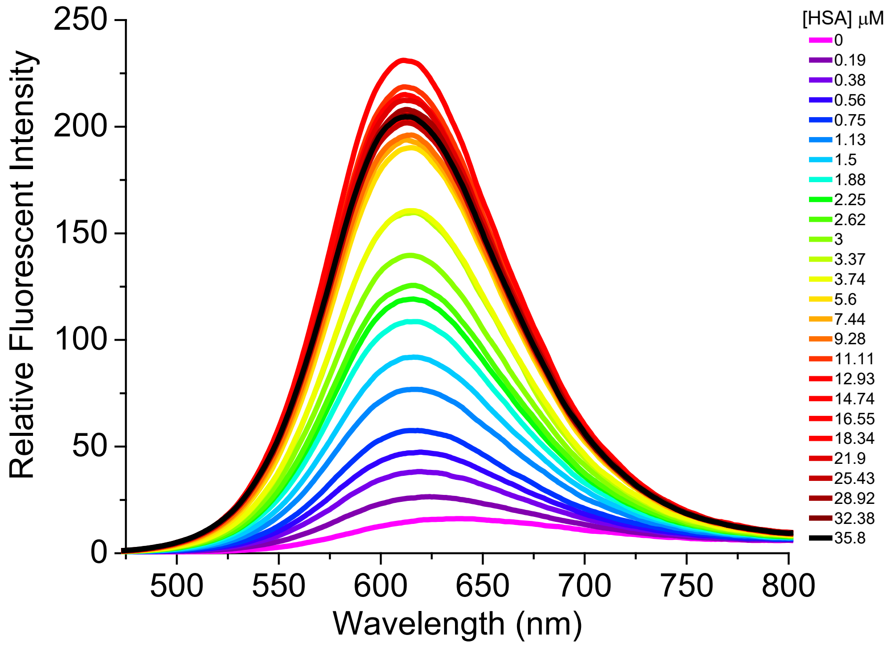

Figure S16. Fluorescent response of sensor **1b** acquired during titration with 5% HSA in PBS.

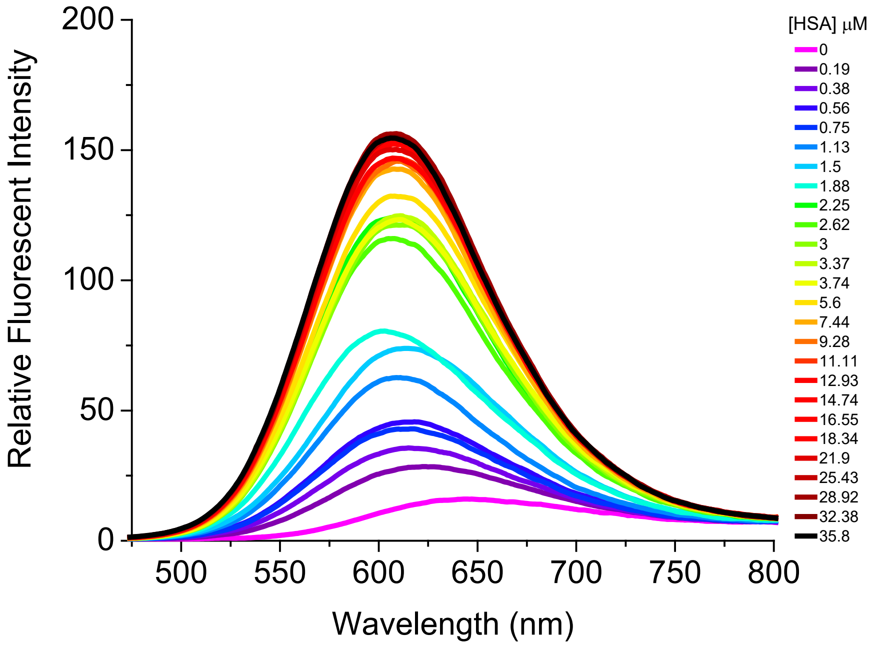

Figure S17. Fluorescent response of sensor **1c** acquired during titration with 5% HSA in PBS.

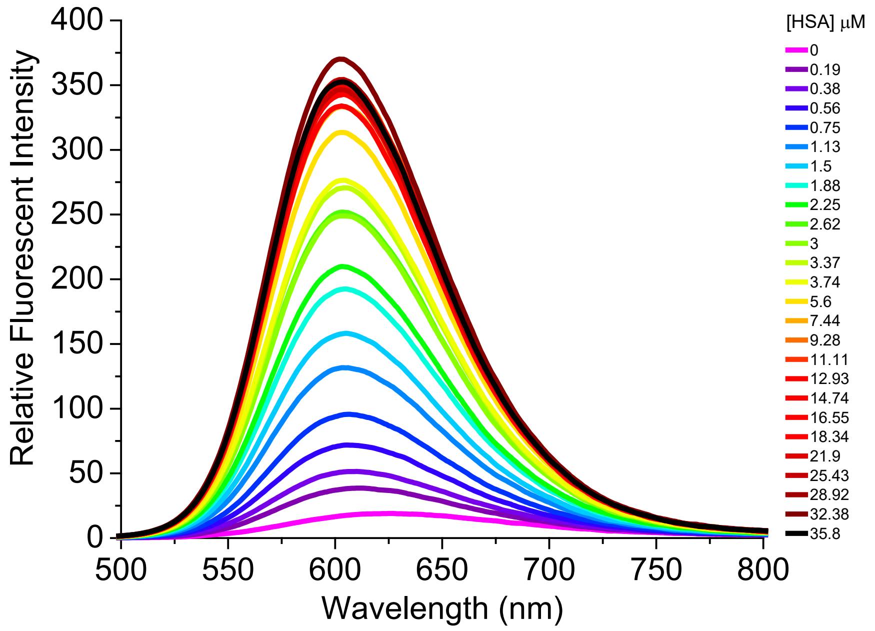

Figure S18. Fluorescent response of sensor **1d** acquired during titration with 5% HSA in PBS.

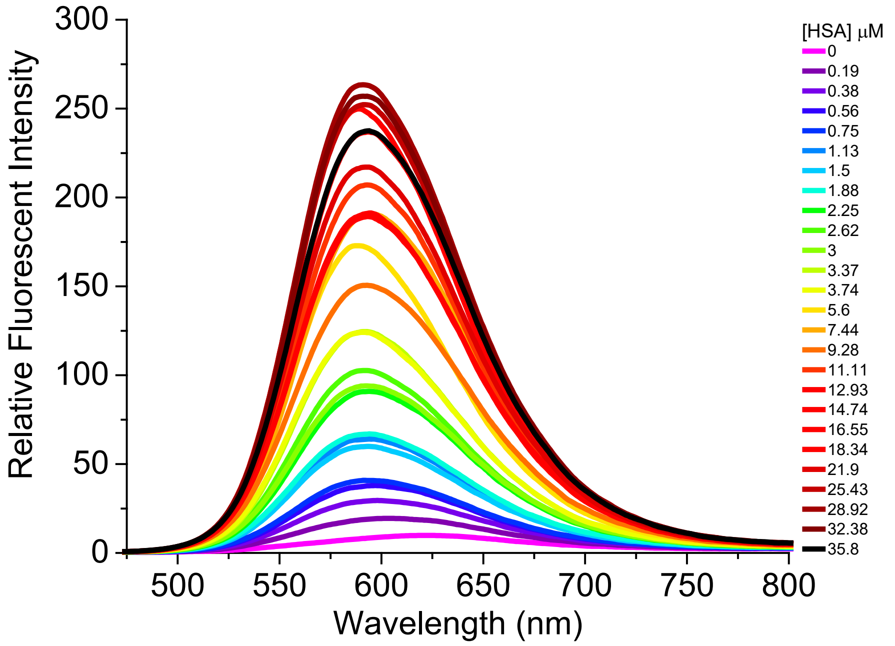

Figure S19. Fluorescent response of sensor **1e** acquired during titration with 5% HSA in PBS.

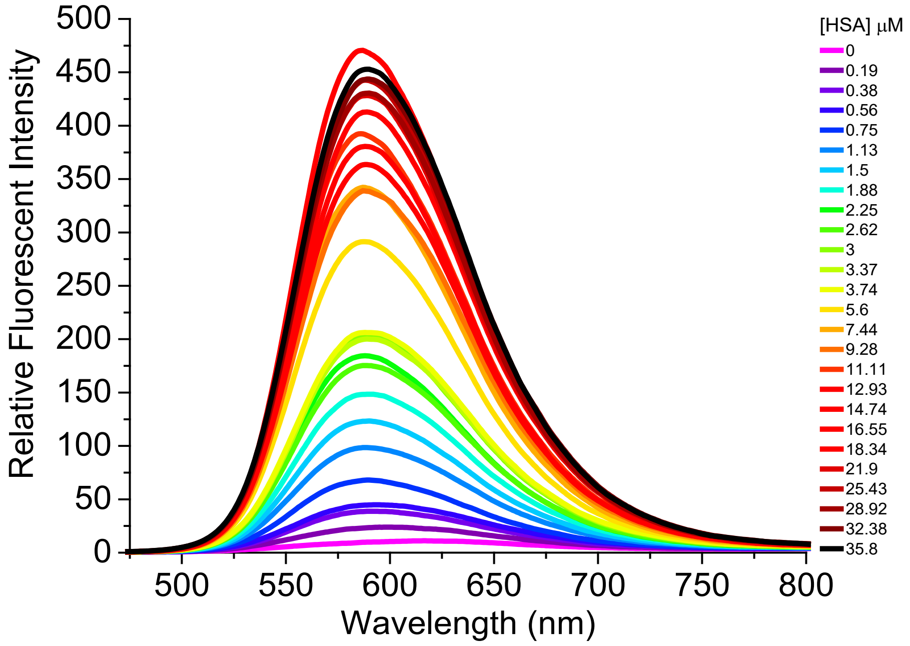

Figure S20. Fluorescent response of sensor **1f** acquired during titration with 5% HSA in PBS.

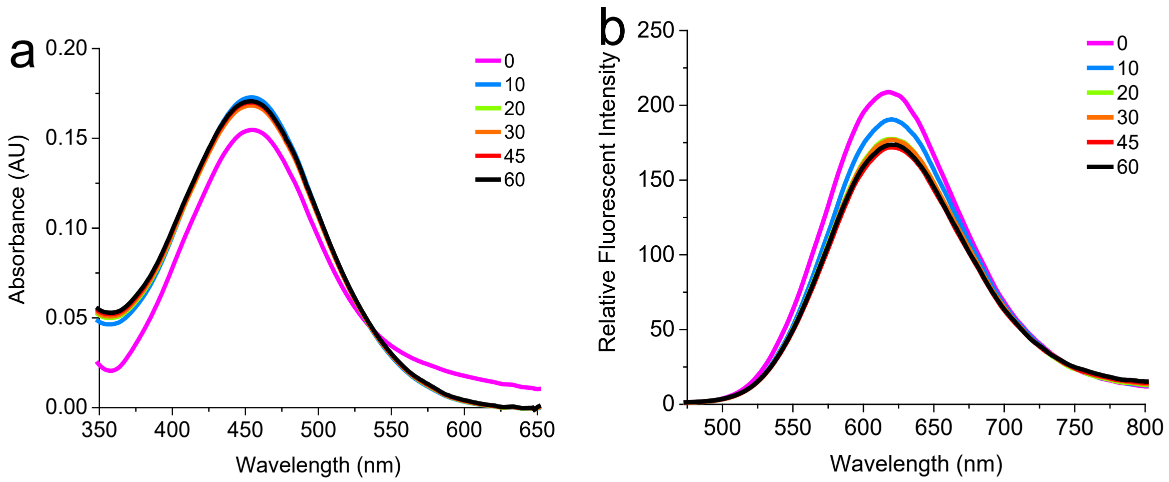

Figure S21. Time-dependent absorption (a) and fluorescence (b) spectra of chalcone **1a** in PBS following addition of HSA (35.8 μM). Spectra were recorded over a 60 min period to monitor the stability and evolution of the protein-bound state.

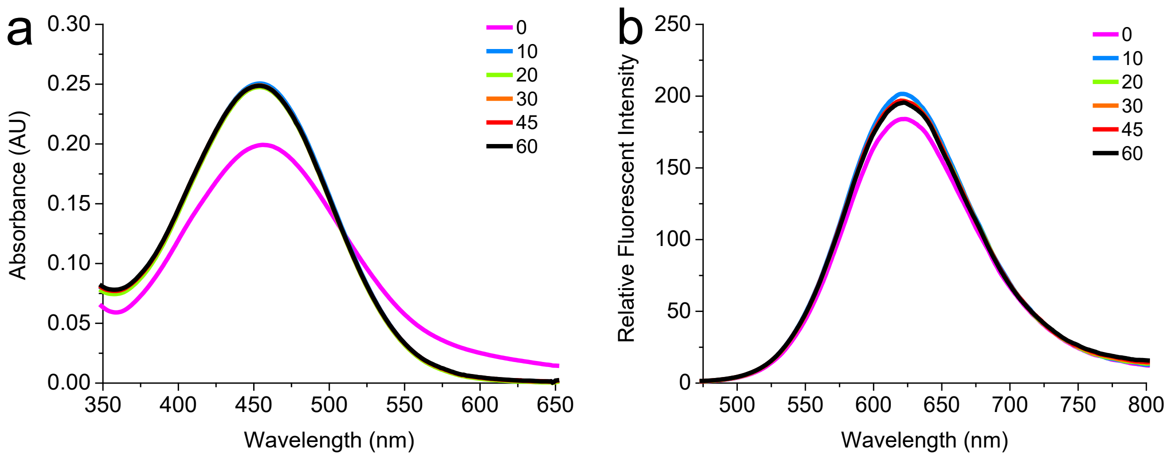

Figure S22. Time-dependent absorption (a) and fluorescence (b) spectra of chalcone **1b** in PBS following addition of HSA (35.8 μM). Spectra were recorded over a 60 min period to monitor the stability and evolution of the protein-bound state.

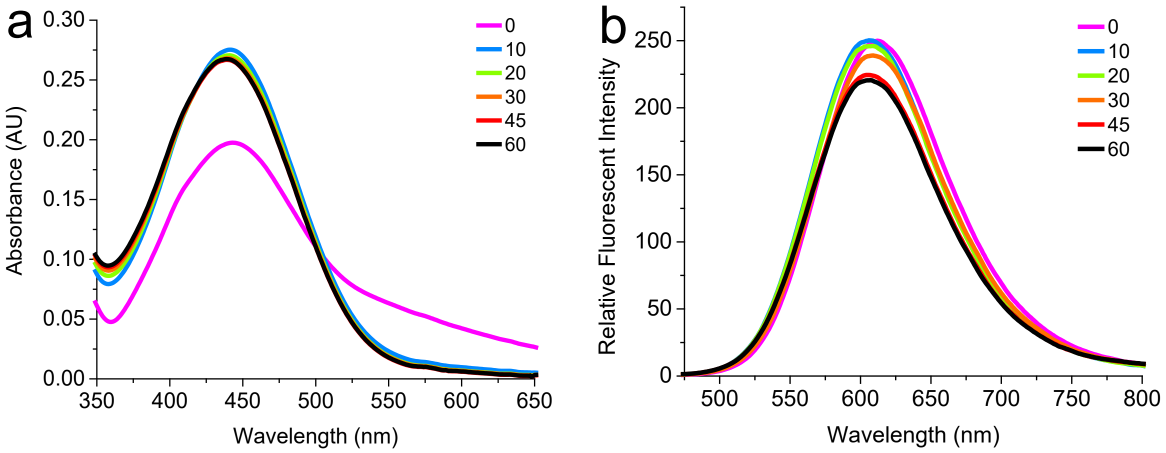

Figure S23. Time-dependent absorption (a) and fluorescence (b) spectra of chalcone **1c** in PBS following addition of HSA (35.8 µM). Spectra were recorded over a 60 min period to monitor the stability and evolution of the protein-bound state.

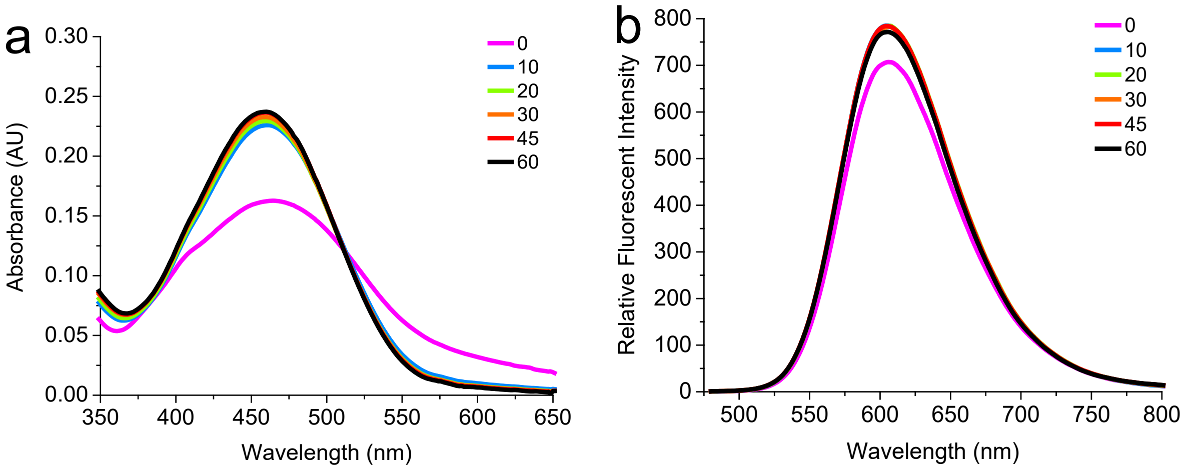

Figure S24. Time-dependent absorption (a) and fluorescence (b) spectra of chalcone **1d** in PBS following addition of HSA (35.8 µM). Spectra were recorded over a 60 min period to monitor the stability and evolution of the protein-bound state.

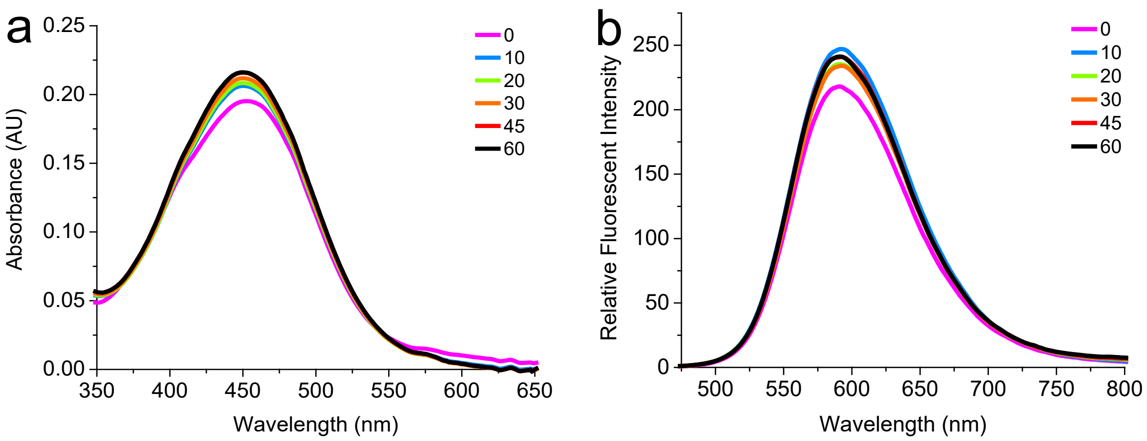

Figure S25. Time-dependent absorption (a) and fluorescence (b) spectra of chalcone **1e** in PBS following addition of HSA (35.8 μM). Spectra were recorded over a 60 min period to monitor the stability and evolution of the protein-bound state.

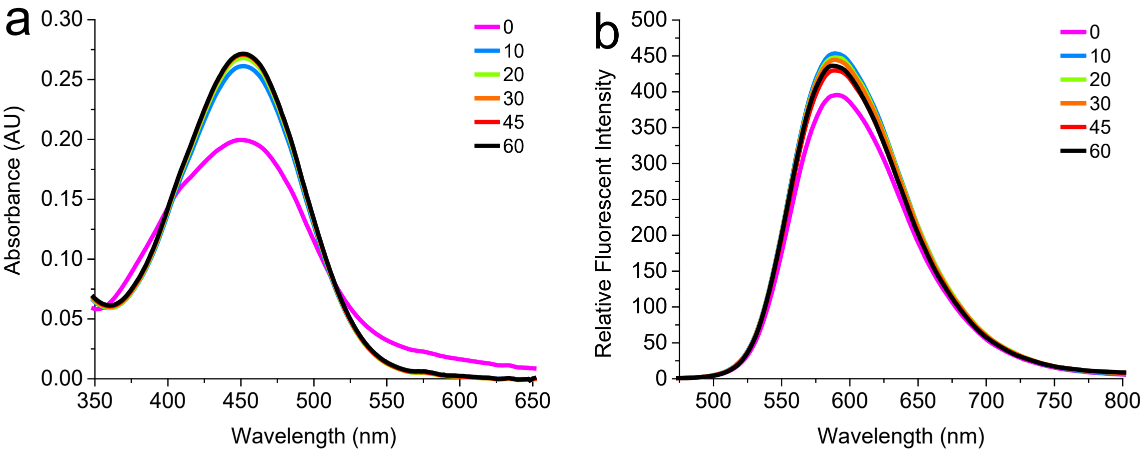

Figure S26. Time-dependent absorption (a) and fluorescence (b) spectra of chalcone **1f** in PBS following addition of HSA (35.8 μM). Spectra were recorded over a 60 min period to monitor the stability and evolution of the protein-bound state.

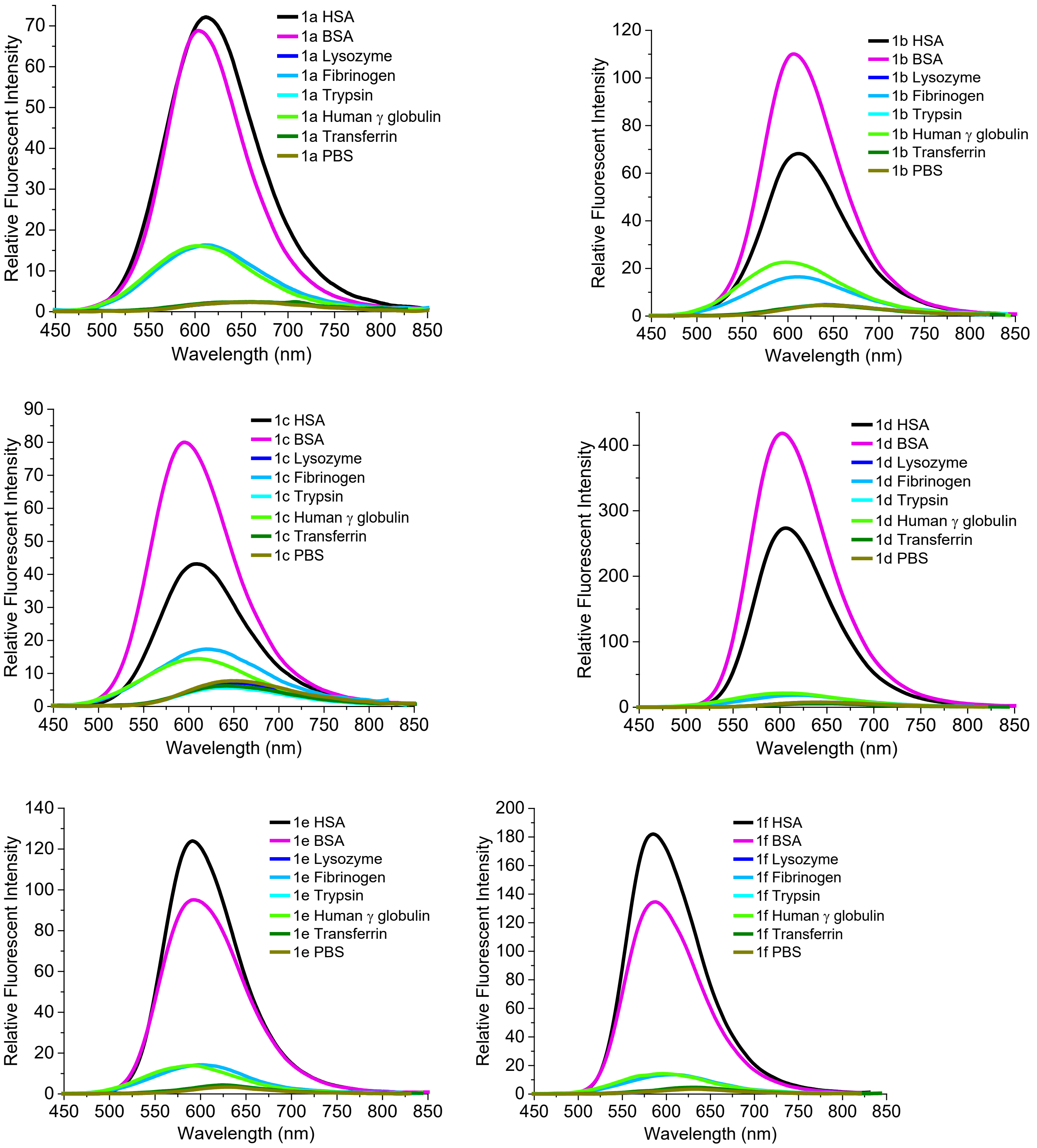

Figure S27. Fluorescence response of chalcone sensors **1a–1f** (2.5 μM) in the presence of HSA, BSA, lysozyme, fibrinogen, and trypsin (5 μM) in PBS (pH 7.4).

Table S2. Docking parameters and residue interactions for chalcone **1a** across the three lowest energy binding poses. Values include binding energy, ligand efficiency, inhibition constant, geometry, contact distances, residues in close contact, and polar interactions.

Parameter	Pose 1	Pose 2	Pose 3
**Binding Energy (kcal/mol)**	–6.11	–5.20	–4.18
**Ligand Efficiency**	–0.29	–0.25	–0.20
**Inhibition Constant (µM)**	33.09	153.94	856.28
**Geometry**	s-cisoid	s-cisoid	s-cisoid
**Binding Site**	Drug site III	Drug site III	Alternate Site
**Contacts (Å)**	—	—	—
**Residues in Close Contact**	Arg186, Tyr161, Tyr138, Leu115, Phe134	Arg186, Leu115, Met123, Phe134, Tyr161, Tyr138, Lys137	—

Table S3. Docking parameters and residue interactions for chalcone **1b** across the three lowest energy binding poses. Values include binding energy, ligand efficiency, inhibition constant, geometry, contact distances, residues in close contact, and polar interactions.

Parameter	Pose 1	Pose 2	Pose 3
**Binding Energy (kcal/mol)**	–7.34	–6.98	–6.90
**Ligand Efficiency**	–0.32	–0.30	–0.30
**Inhibition Constant (µM)**	4.15	7.62	8.68
**Geometry**	s-cisoid	s-cisoid	s-cisoid
**Binding Site**	Drug site III	Drug site III	Drug site III
**Contacts (Å)**	—	—	—
**Residues in Close Contact**	Leu115, Arg117, Leu182, Tyr138, Met123, Phe134, Lys137	Arg117, Leu115, Lys137, Ile142, Phe134, Met123, Tyr161	Lys137, Leu115, Arg186, Tyr138, Tyr161, Arg117, Phe134, Met123, Ala126

Table S4. Docking parameters and residue interactions for chalcone **1c** across the three lowest energy binding poses. Values include binding energy, ligand efficiency, inhibition constant, geometry, contact distances, residues in close contact, and polar interactions.

Parameter	Pose 1	Pose 2	Pose 3
**Binding Energy (kcal/mol)**	−7.67	−7.67	−7.41
**Ligand Efficiency**	−0.33	−0.33	−0.32
**Inhibition Constant (μM)**	2.37	2.38	3.69
**Geometry**	s-transoid	s-transoid	s-cisoid
**Binding Site**	Drug site III	Drug site III	Drug site III
**Contacts (Å)**	DMA–Lys137: 3.782; 4-OMe–Arg117: 5.238	DMA–Lys137: 4.084; 4-OMe–Arg117: 5.280	—
**Residues in Close Contact**	Arg186, Arg117, Leu182, Leu185, Tyr161, Tyr138, Met123, Phe134, Lys137	Leu185, Leu182, Arg117, Lys137, Met123, Phe134, Tyr138	Leu185, Leu182, Arg117, Leu115, Tyr161, Tyr138, Phe134, Ala126, Met123, Lys137

Table S5. Docking parameters and residue interactions for chalcone **1d** across the three lowest energy binding poses. Values include binding energy, ligand efficiency, inhibition constant, geometry, contact distances, residues in close contact, and polar interactions.

Parameter	Pose 1	Pose 2	Pose 3
**Binding Energy (kcal/mol)**	−7.48	−7.24	−7.10
**Ligand Efficiency**	−0.30	−0.29	−0.28
**Inhibition Constant (μM)**	3.3	4.94	6.2
**Geometry**	s-cisoid	s-cisoid	s-cisoid
**Binding Site**	Drug site III	Drug site III	Drug site III
**Contacts (Å)**	DMA–Lys137: 3.389; 4-OMe–Arg117: 3.203	DMA–Lys137: 4.677	Carbonyl–Tyr161 H-bond: 2.8
**Residues in Close Contact**	Leu115, Arg117, Lys137, Phe134, Tyr161, Tyr138	Phe134, Met123, Lys137, Ile142, Gly189, Arg186	Tyr138, Ile142, Leu115, Phe134, Met123

Table S6. Docking parameters and residue interactions for chalcone **1e** across the three lowest energy binding poses. Values include binding energy, ligand efficiency, inhibition constant, geometry, contact distances, residues in close contact, and polar interactions.

Parameter	Pose 1	Pose 2	Pose 3
**Binding Energy (kcal/mol)**	−7.26	−6.76	−6.38
**Ligand Efficiency**	−0.29	−0.27	−0.26
**Inhibition Constant (μM)**	4.74	11.02	20.93
**Geometry**	s-cisoid	s-cisoid	s-cisoid
**Binding Site**	Drug site III	Drug site III	Alternate Site
**Contacts (Å)**	DMA–Lys137: 3.920; 2-OMe–Arg117: 2.825	DMA–Lys137: 4.090; 2-OMe–Arg117: 2.271	—
**Residues in Close Contact**	Lys137, Phe134, Ile142, Arg186, Leu182, Tyr161	Tyr138, Tyr161, Arg117, Leu115, Phe134, Lys137	—

Table S7. Docking parameters and residue interactions for chalcone **1f** across the three lowest energy binding poses. Values include binding energy, ligand efficiency, inhibition constant, geometry, contact distances, residues in close contact, and polar interactions.

Parameter	Pose 1	Pose 2	Pose 3
**Binding Energy (kcal/mol)**	−7.01	−6.89	−4.54
**Ligand Efficiency**	−0.26	−0.26	−0.17
**Inhibition Constant (μM)**	7.23	8.87	473.71
**Geometry**	s-cisoid	s-cisoid	s-cisoid
**Binding Site**	Drug site III	Drug site III	Alternate site
**Contacts (Å)**	DMA–Lys137: 4.460; Carbonyl–Arg117: 2.269	DMA–Lys137: 4.530; 2-OMe–Arg117: 2.527	—
**Residues in Close Contact**	Arg117, Tyr138, Leu115, Phe134, Met123, Lys137	Ala126, Phe134, Met123, Lys137, Leu115, Arg117, Leu182	—

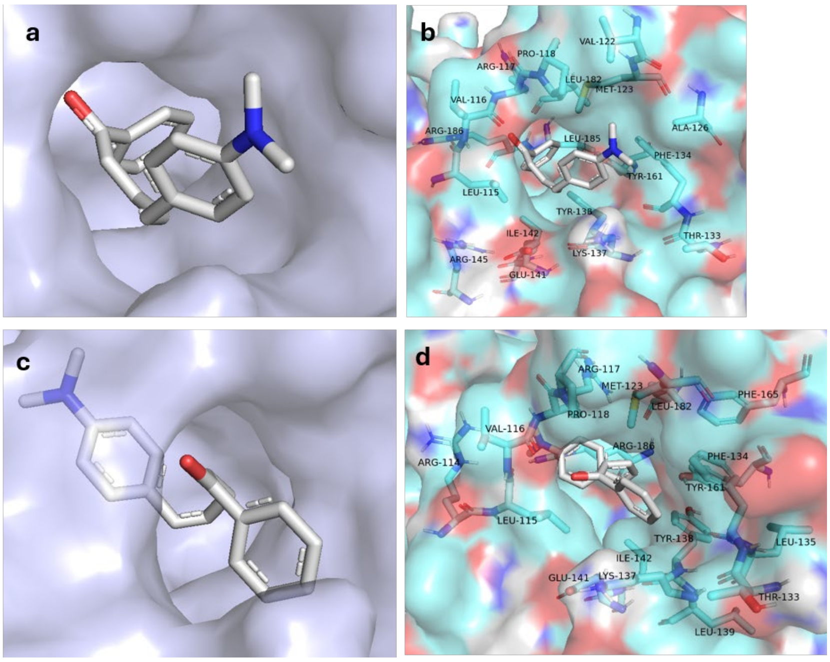

Figure S28. Representative molecular docking poses of chalcone **1a** within the HSA hemin-binding pocket (FA1). Panels **(a,b)** show the lowest-energy docking pose (Pose 1), panels **(c,d)** show the second-ranked docking pose (Pose 2). For each pose, the left panel depicts the ligand within the protein surface, while the right panel highlights the surrounding binding pocket residues. Corresponding binding energies are provided in Table S2.

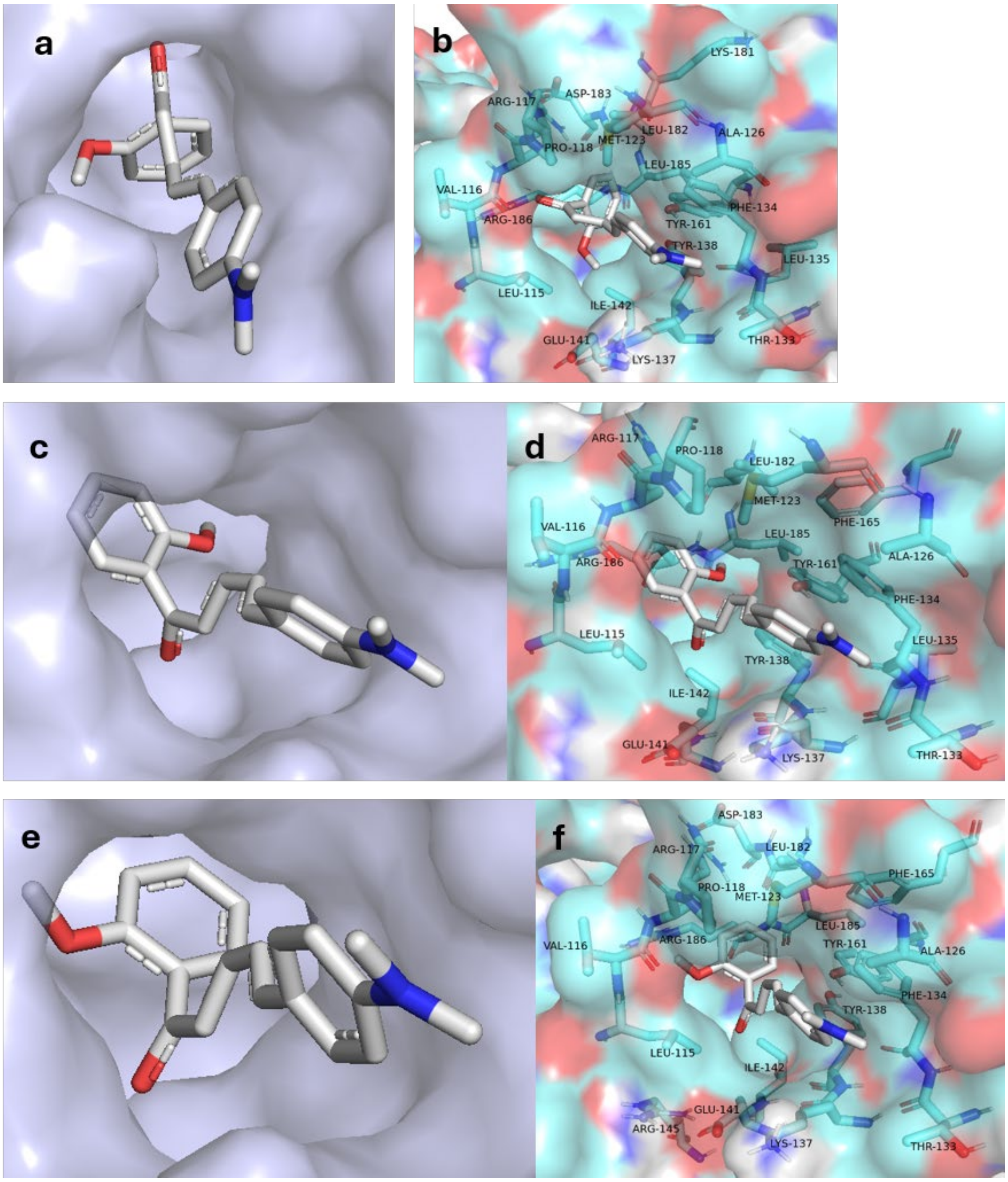

Figure S29. Representative molecular docking poses of chalcone **1b** within the HSA hemin-binding pocket (FA1). Panels **(a,b)** show the lowest-energy docking pose (Pose 1), panels **(c,d)** show the second-ranked docking pose (Pose 2), and panels **(e,f)** show the third-ranked docking pose (Pose 3). For each pose, the left panel depicts the ligand within the protein surface, while the right panel highlights the surrounding binding pocket residues. Corresponding binding energies are provided in Table S3.

a

c

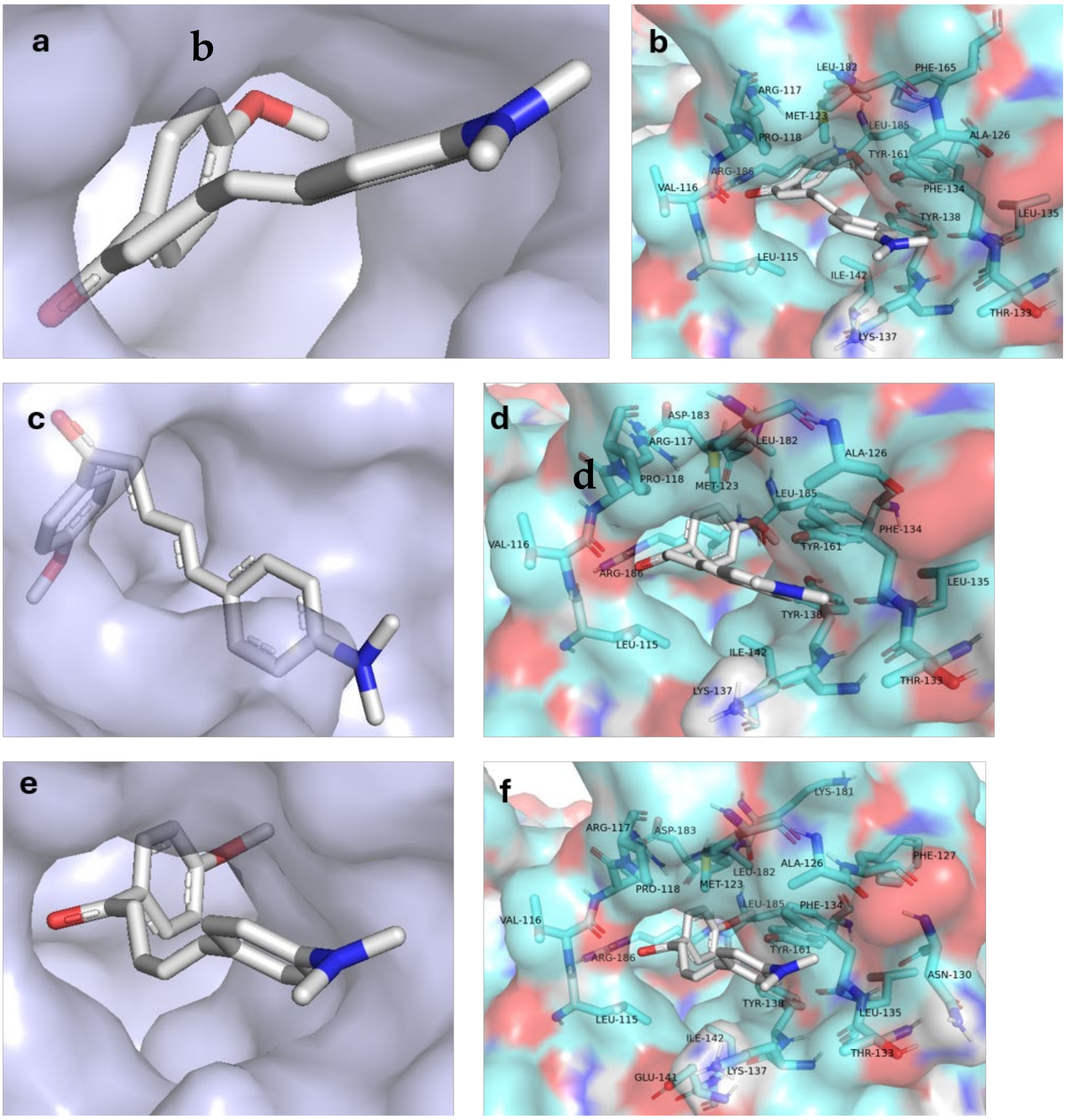

Figure S30. Representative molecular docking poses of chalcone **1c** within the HSA hemin-binding pocket (FA1). Panels **(a,b)** show the lowest-energy docking pose (Pose 1), panels **(c,d)** show the second-ranked docking pose (Pose 2), and panels **(e,f)** show the third-ranked docking pose (Pose 3). For each pose, the left panel depicts the ligand within the protein surface, while the right panel highlights the surrounding binding pocket residues. Corresponding binding energies are provided in Table S4.

a

c

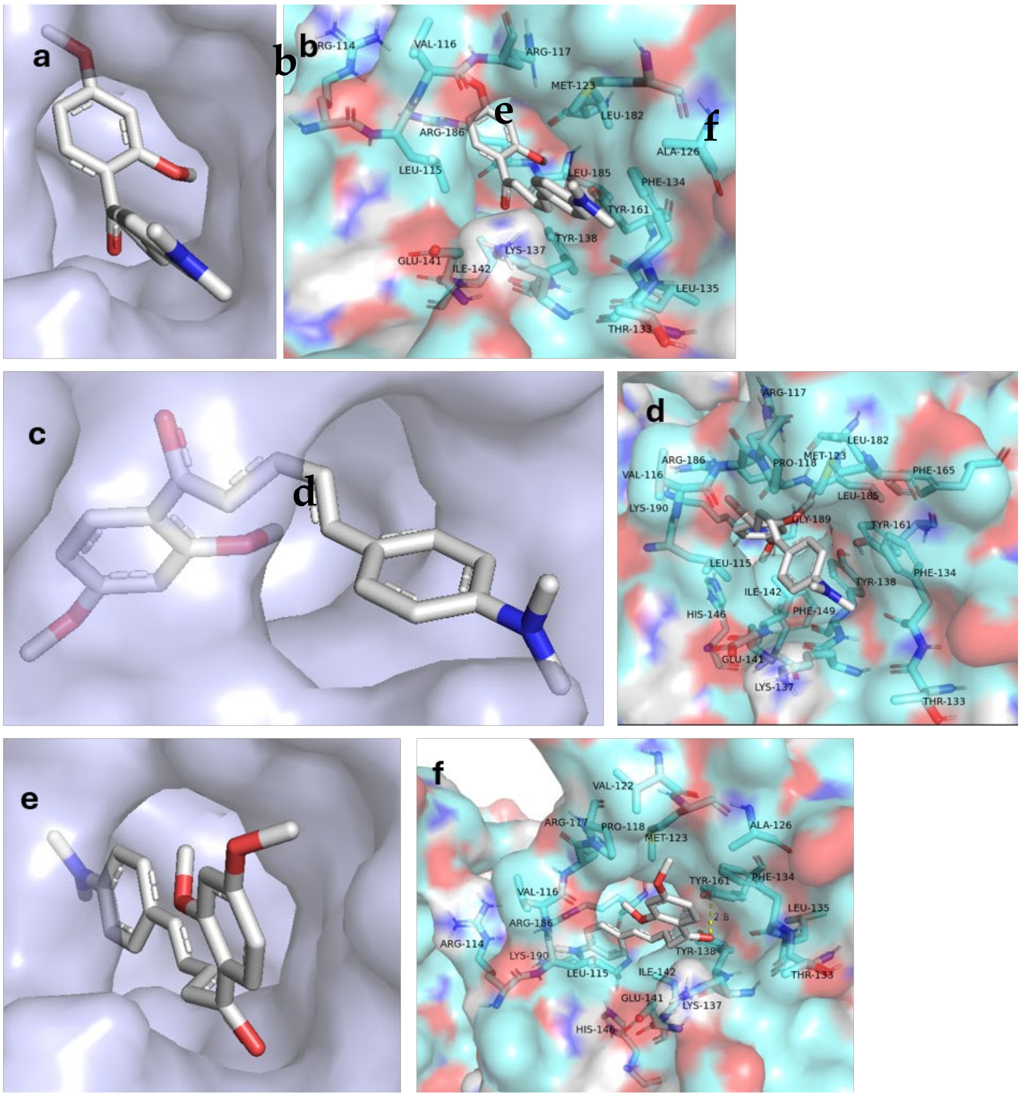

Figure S31. Representative molecular docking poses of chalcone **1d** within the HSA hemin-binding pocket (FA1). Panels **(a,b)** show the lowest-energy docking pose (Pose 1), panels **(c,d)** show the second-ranked docking pose (Pose 2), and panels **(e,f)** show the third-ranked docking pose (Pose 3). For each pose, the left panel depicts the ligand within the protein surface, while the right panel highlights the surrounding binding pocket residues. Corresponding binding energies are provided in Table S5.

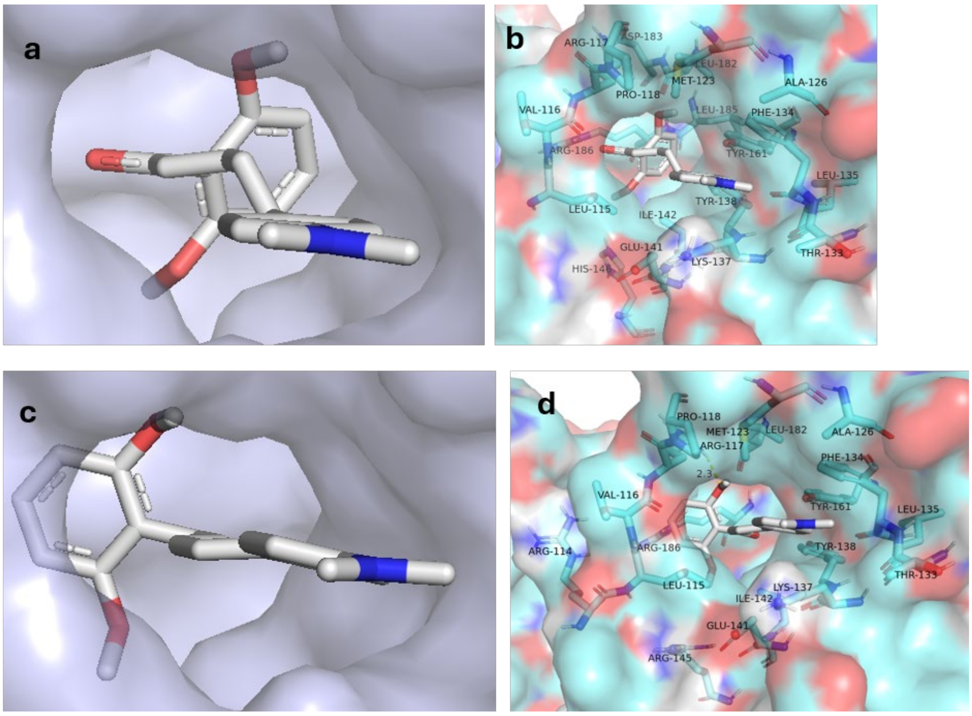

Figure S32. Representative molecular docking poses of chalcone **1e** within the HSA hemin-binding pocket (FA1). Panels **(a,b)** show the lowest-energy docking pose (Pose 1), panels **(c,d)** show the second-ranked docking pose (Pose 2). For each pose, the left panel depicts the ligand within the protein surface, while the right panel highlights the surrounding binding pocket residues. Corresponding binding energies are provided in Table S6.

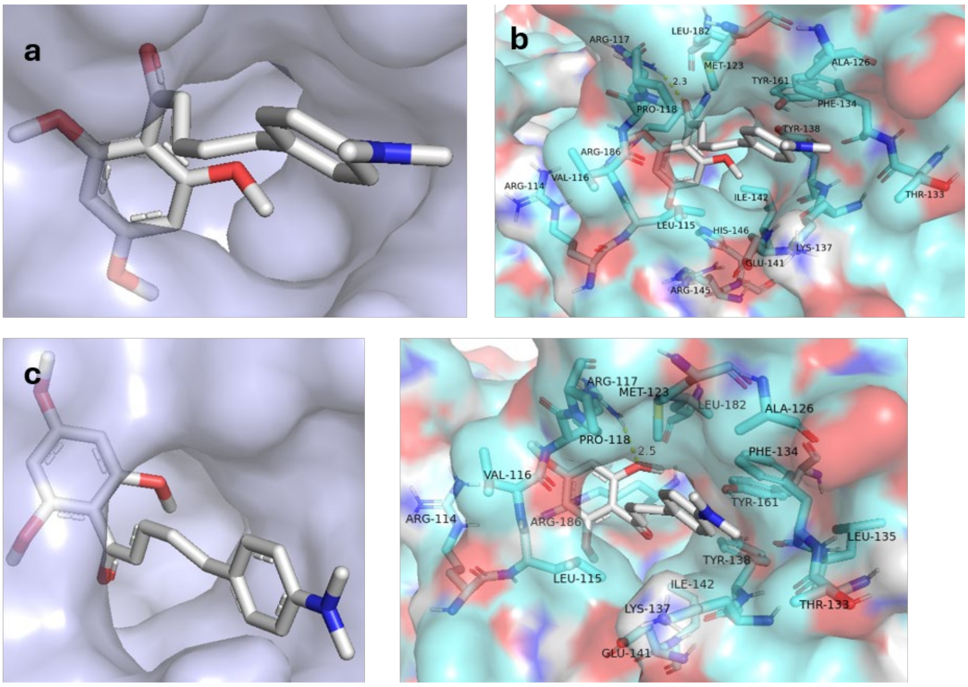

Figure S33. Representative molecular docking poses of chalcone **1f** within the HSA hemin-binding pocket (FA1). Panels **(a,b)** show the lowest-energy docking pose (Pose 1), panels **(c,d)** show the second-ranked docking pose (Pose 2). For each pose, the left panel depicts the ligand within the protein surface, while the right panel highlights the surrounding binding pocket residues. Corresponding binding energies are provided in Table S7.
